# *Parabacteroides distasonis* regulates the infectivity and pathogenicity of SVCV at different water temperatures

**DOI:** 10.1186/s40168-024-01799-9

**Published:** 2024-07-17

**Authors:** Yujun Zhang, Yan Gao, Chen Li, Yong-An Zhang, Yuanan Lu, Jing Ye, Xueqin Liu

**Affiliations:** 1 National Key Laboratory of Agricultural Microbiology, College of Fisheries, Huazhong Agricultural University, Wuhan, Hubei China; 2 Hubei Engineering Technology Research Center for Aquatic Animal Diseases Control and Prevention, Wuhan, Hubei China; 3 Ocean College, Hebei Agricultural University, Qinhuangdao, Hebei China; 4 Department of Public Health Sciences, Thompson School of Social Work & Public Health, University of Hawaii at Manoa, Honolulu, HI USA; 5 National Key Laboratory of Agricultural Microbiology, College of Veterinary Medicine, Huazhong Agricultural University, Wuhan, Hubei China

**Keywords:** Spring viremia of carp virus, Temperature, *Parabacteroides distasonis*, Deoxycholic acid, Zebrafish

## Abstract

**Background:**

Spring viremia of carp virus (SVCV) infects a wide range of fish species and causes high mortality rates in aquaculture. This viral infection is characterized by seasonal outbreaks that are temperature-dependent. However, the specific mechanism behind temperature-dependent SVCV infectivity and pathogenicity remains unclear. Given the high sensitivity of the composition of intestinal microbiota to temperature changes, it would be interesting to investigate if the intestinal microbiota of fish could play a role in modulating the infectivity of SVCV at different temperatures.

**Results:**

Our study found that significantly higher infectivity and pathogenicity of SVCV infection in zebrafish occurred at relatively lower temperature. Comparative analysis of the intestinal microbiota in zebrafish exposed to high- and low-temperature conditions revealed that temperature influenced the abundance and diversity of the intestinal microbiota in zebrafish. A significantly higher abundance of *Parabacteroides distasonis* and its metabolite secondary bile acid (deoxycholic acid, DCA) was detected in the intestine of zebrafish exposed to high temperature. Both colonization of *Parabacteroides distasonis* and feeding of DCA to zebrafish at low temperature significantly reduced the mortality caused by SVCV. An in vitro assay demonstrated that DCA could inhibit the assembly and release of SVCV. Notably, DCA also showed an inhibitory effect on the infectious hematopoietic necrosis virus, another *Rhabdoviridae* member known to be more infectious at low temperature.

**Conclusions:**

This study provides evidence that temperature can be an important factor to influence the composition of intestinal microbiota in zebrafish, consequently impacting the infectivity and pathogenicity of SVCV. The findings highlight the enrichment of *Parabacteroides distasonis* and its derivative, DCA, in the intestines of zebrafish raised at high temperature, and they possess an important role in preventing the infection of SVCV and other *Rhabdoviridae* members in host fish.

Video Abstract

**Supplementary Information:**

The online version contains supplementary material available at 10.1186/s40168-024-01799-9.

## Introduction

The majority of teleost fish are poikilothermic, meaning they are unable to regulate their body temperature and are therefore vulnerable to fluctuations in water temperature since they are unable to regulate their body temperature. These fluctuations can have a significant impact on the prevalence of viral diseases among fish populations [1]. In the aquaculture industry, viruses pose the greatest threat to the health of aquatic animals. The World Organization for Animal Health listed 10 notable diseases in the 2022 Aquatic Animal Health Code, with eight of them being viral diseases (https://www.woah.org/). Most of these viruses have a significant dependence on temperature for their replication. Currently, there is no effective commercial method for combating viral infections [2]. Vaccine exploitation in aquaculture has primarily focused on bacterial pathogens, with limited progress made in vaccine control of aquatic viruses. The optimal temperature for viral infection varies depending on the types of aquatic viruses. For instance, Cyprinid herpesvirus 3 (CyHV-3) is known to cause the disease at temperatures ranging from 18 to 28 °C [3]. Hirame rhabdovirus virus (HIRRV) causes high mortality rates in both marine and freshwater fish with peak morbidity at water temperatures around 10 °C and but less morbidity when water temperatures are above 20 °C [4, 5]. The seasonal epidemic of viruses can evade host immune surveillance, facilitating virus transmission. Prompt and effective treatment is crucial for viral disease management, and an improved understanding of the temperature-dependent infectivity of aquatic viruses can form an essential basis for developing broad-spectrum treatments for these viruses.

Spring viremia of carp virus (SVCV) is a member of the genus *Sprivivirus*, family *Rhabdoviridae* containing a non-segmented single-strand negative RNA genome [6]. The virus infects a wide range of fish species with a particular target to cyprinids, such as common carp (*Cyprinus carpio*). SVCV is prevalent in various regions across the Americas, Europe, and Asia, posing a serious threat to the aquaculture industry due to its high mortality rate in juvenile fish [7]. SVCV causes Spring viremia of carp (SVC), leading to symptoms such as internal bleeding, abdominal inflammation, nervous disorders, and hydrocephalus [8, 9]. Currently, there have been no drugs or vaccines available for SVC management. The pathogenesis of SVCV primarily occurs during spring when the water temperature is below 20 °C, but SVCV incidence decreases significantly as the temperature rises to 28 °C [10]. A comparison of the expression of MAVS and IRF7 in ZFL cells at 16 °C and 28 °C showed that the elevated temperature increased the expression of the IFN system [11]. This increase could explain the high-temperature suppression of SVCV. However, it is worth noting that different viruses can infect the same species of fish at distinct temperatures in aquaculture [12, 13]. Therefore, the role of innate immunity in determining viral infectivity based on temperature remains inconclusive. Understanding the pathogenesis associated with temperature can provide valuable insights and approaches for controlling and preventing SVCV, as well as other fish diseases.

There is increasing evidence that the intestinal microbiota is sensitive to environmental and internal temperatures [14–16] and plays a key role in regulating host immunity and resisting viral infections [17–20]. Pair-feeding experiments verified that hypothermia caused changes in the intestinal microbiota rather than the amount of food intake, and further verified that an increase of host norepinephrine in turn regulated the composition of the intestinal microbiota [21]. Poikilothermic organisms such as teleost fish experience temperature fluctuations that are absent in homeotherms, making their gut microbiomes more susceptible to temperature changes [22–24]. Seasonal changes in water temperature can significantly impact the gut microbiomes of teleost fish [25]. For instance, a study on gut microbiomes of blue tilapia at 12 °C and 24 °C suggested that low-temperature conditions are highly selective and may constrain the microbial community and reduce its inter-individual variability [26]. A recent report has indicated that acute heat stress can reduce the relative abundance and diversity of intestinal flora in rainbow trout and cause changes in serum amino acids, vitamins, and short-chain fatty acid metabolites [27]. There is a potential relationship between temperature-induced alterations in intestinal microbiota and their effects on the infectivity and pathogenicity of viruses.

In this study, we demonstrated that the composition of intestinal microbiota is associated with the temperature-dependent infectivity of SVCV. Our results suggest that *Parabacteroides distasonis*, which is abundantly found in the intestine of zebrafish kept at high temperature (HT), plays a critical role in restricting SVCV infection. Its metabolite, deoxycholic acid (DCA), showed potent suppressive effects on the replication and pathogenicity of SVCV and other viral members in the *Rhabdoviridae* family that cause diseases at low temperature (LT). This study not only improves the comprehension of the pathogenesis of SVCV regulated by temperature but also provides new insight into the development of anti-SVCV probiotics and pharmaceutical interventions.

## Materials and methods

### Fish, cell lines, viruses, and bacteria

The AB zebrafish (*Danio rerio*) were obtained from the China Zebrafish Resource Center (CZRC). The rainbow trout were purchased from a farm in Sichuan and placed in a breeding aquarium at 16 °C for adaptation. The epithelioma papulosum cyprini (EPC) cell line (ATCC CRL-2872) was maintained at 28 °C in MEM (Hyclone) supplemented with 10% fetal bovine serum (Life Technologies). SVCV (ATCC VR-1390) was propagated in EPC cells at 28 °C and harvested when more than 80% virus-induced cytopathic effect appeared. IHNV was propagated in EPC cells at 16 °C and harvested when virus-induced cytopathic effect appeared. *Parabacteroides distasonis* (ATCC 8503) was cultured in 50 mL of YCFA medium at 28 °C in an anaerobic chamber for 24 h. Cell pellets were obtained by centrifugation at 8000 *g* for 10 min at 4 °C. To prepare the cell suspension for oral administration, the cultured bacterial cells were suspended in oxygen-free PBS, resulting in a final cell density of 1 × 10^9^ colony-forming units (CFU) per mL.

### Chemicals and reagents

Chemical reagents were sourced from Sigma-Aldrich (St. Louis, MO, USA), Yuanye (Shanghai, China), MedChemExpress (New Jersey, USA), and Aladdin (Shanghai, China). Ampicillin, streptomycin, vancomycin, and colistin were obtained from Yuanye. Cholestyramine (CHO) was obtained from MCE. DCA was obtained from Aladdin, 7-ketolithocholic acid (7-ketoLCA) from MCE, and 7-ketodeoxycholic acid (7-ketoDCA) from Sigma-Aldrich. These bile acids (BAs) were accurately weighed and prepared in dimethyl sulfoxide (DMSO) to obtain individual stock solutions of 100 mM.

### Zebrafish breeding

AB zebrafish were initially reared in an aquarium at 28 ± 1 °C for adaptation. To enable SVCV infection in zebrafish, the tank water temperature was gradually reduced from 28 to 16 °C at a rate of 1 °C per day.

### Antibiotic treatment

The zebrafish bred in high or low water temperature were treated with an antibiotic regimen following the method outlined in a previous study [28]. Briefly, a mixture of streptomycin (2 mg/L), ampicillin (2 mg/L), colistin (2 mg/L), and vancomycin (1 mg/L) was added to the water where zebrafish were housed for 7 days. Additionally, zebrafish were fed a combination (5 ml) of antibiotics including streptomycin (0.2 mg/mL), ampicillin (0.2 mg/mL), colistin (0.2 mg/mL), and vancomycin (0.1 mg/mL), along with artemia, as food twice a day for 7 days. The antibiotics (ABX) treatment was completed 2 days prior to microbiota transplantation.

### Bacterial colonization

For intestinal microbiota transplantation (IMT), the intestines of zebrafish at HT (28 °C) were collected using the established methods [29, 30]. The collected samples were immediately homogenized in a sterile N^2^-filled environment and filtered through a 0.25-mm stainless steel filter to collect bacteria, followed by centrifugation at 6000 × *g* for 15 min. The samples were then either used directly for IMT or stored at − 80 °C after resuspension in PBS containing 10% (v/v) glycerol. Prior to IMT, samples were centrifuged to replace glycerol to PBS. The number of viable microbes was determined using optical microscopy with methylene blue staining. Zebrafish which have been treated with ABX for 7 days (see the method above) were subjected to IMT 2 days after completing ABX treatment. IMT was conducted by orally feeding 100 μL of a 10^8^ CFU bacteria suspension or PBS (negative control) to the zebrafish. Subsequently, all zebrafish were intraperitoneally injected with 10^4^ PFU of SVCV, and the infected fish were observed for 14 days following the infection. The schematic representations of the procedures are shown in Fig. 2a and c.

To colonize *Parabacteroides distasonis* in zebrafish living at LT (16 °C), the zebrafish were randomly assigned to two groups. One group was administered a daily oral gavage of sterile PBS (100 μL) as a negative control, while the other group received a suspension of *Parabacteroides distasonis* (100 μL). After 3 days of oral administration, the zebrafish were moved to fresh water for an additional day. Following this, all treated zebrafish were intraperitoneally injected with either 10^4^ PFU of SVCV suspension (10 μL) or equal volume of M199 medium. Infected fish were then monitored for 14 days post-infection. The schematic representation of the procedure is shown in Fig. 4a.

### High-throughput sequencing of the 16S rRNA gene

Intestines of zebrafish from HT and LT environments, as well as those treated with ABX or ABX + IMT, were collected for 16S rRNA sequencing, with each group consisting of 4 replicates and each replicate comprising three individuals. The samples were homogenized, and total DNA was extracted using the CTAB method. According to the concentration, DNA was diluted to 1 ng/μL using sterile water. The 16S rRNA gene V4 region primer pair of 515F (5′-GTGCCAGCMGCCGCGGTAA-3′) and 806R (5′-GGACTACHVGGGTWTCTAAT-3′) was used to amplify the gene. The primer pair was modified with a barcode tag that contained a random 6-base oligo. In order to generate sequencing libraries, the TruSeq DNA PCR-Free Sample Preparation Kit (Illumina, USA) was utilized. The quantity of the library was determined using the Qubit 2.0 Fluorometer (Thermo Scientific, USA). The libraries were then sequenced using the Hiseq2500 platform (Illumina, USA) at Novogene Bioinformatics Technology Co., Ltd. The 250-bp paired-end reads of raw data were obtained from the Hiseq2500 platform.

Paired-end reads were merged to obtain the splicing sequences using FLASH (V1.2.7, http://ccb.jhu.edu/software/FLASH/) [31]. To remove the barcode and primer sequence, the sequences were processed using QIIME (Version 1.7.0) [32]. QIIME was also used to filter out raw tags with low quality to ensure sequencing quality. The tags were compared to the Gold database (Version 7) [33] using the UCHIME algorithm, which helped in removing chimera sequences and retrieving effective reads.

Sequences with a similarity of more than 97% were assigned to the same operational taxonomic unit (OTU) [34]. The GreenGene Database (Version gg_13_5) was used as the reference database to align the sequences, and taxonomic information was annotated using the RDP classifier (Version 2.2) with a confidence threshold of 80% [35]. Alpha diversity metrics, such as the Shannon, Simpson, and Chao1 indices, were calculated using QIIME (Version 1.7.0) and visualized using R software (Version 2.15.3).

### Untargeted metabolomics of the zebrafish intestine

Intestines of zebrafish from HT and LT environments were collected for metabolomic analysis, with each group consisting of 4 replicates and each replicate comprising three individuals. The zebrafish intestines (100 mg) were individually ground with liquid nitrogen. The resulting homogenates were then resuspended with prechilled 80% methanol through vigorous vortexing. After incubating the samples on ice for 5 min, they were centrifuged at 4 °C and 15,000*g* for 20 min. Some of the supernatant was diluted to a final concentration containing 53% methanol by LC–MS grade water. The samples were transferred to new Eppendorf tubes and centrifuged again at 4 °C and 15,000*g* for 20 min. In the end, the supernatants were injected into the liquid chromatography-mass spectrometry (LC–MS/MS) system for analysis.

The identification of these metabolites was performed using the Human Metabolome Database (HMDB, https://hmdb.ca/metabolites), Kyoto Encyclopedia of Genes and Genomes (KEGG) database (https://www.genome.jp/kegg/pathway.html), and LIPIDMaps database (http://www.lipidmaps.org/). Principal components analysis (PCA) was conducted using metaX, a versatile and comprehensive software for processing metabolomics data. Univariate analysis (*t*-test) was conducted to determine the statistical significance (*P*-value). Metabolites were considered differential if they had a VIP > 1, *P*-value < 0.05, and fold change ≥ 2 or FC ≤ 0.5. Volcano plots, generated using ggplot2 in the R language, were used to identify metabolites of interest based on the log2 (fold change) and -log10 (*p*-value) values.

### Cholestyramine administration

Five milligrams of CHO was diluted in 50 μl of DMSO and then mixed with a 5-ml liquid solution of artemia. An equal volume of DMSO mixed with artemia was used as a control. The mixture was used as food for zebrafish twice daily over a period of 7 days. Following the completion of cholestyramine administration, the zebrafish were intraperitoneally injected with 10^4^ PFU of SVCV 1 day later. The schematic representation of the procedure is shown in Fig. 5a.

### Bile acid administration

For bile acid administration in zebrafish, individual bile acid was diluted in 50 μl of DMSO and then mixed with a 5-ml liquid solution of artemia to prepare a 1-mM bile acid mixture. An equal volume of DMSO mixed with artemia was used as a control. The mixture was used as food for zebrafish twice daily for a period of 14 days. Following the completion of bile acid administration, zebrafish were intraperitoneally injected with 10^4^ PFU of SVCV 1 day later. The schematic representation of the procedure is shown in Fig. 5d.

For DCA administration in rainbow trout, 100 mM DCA (50 μl) was added to 5-g rainbow trout diet and mixed homogenously. An equivalent volume of DMSO mixed with diet was served as the control. The rainbow trout were fed the mixed diet as food twice daily for a period of 14 days. Following the completion of DCA administration, rainbow trout were intraperitoneally injected with 10^4^ PFU of IHNV 1 day later.

### Quantitative real-time PCR (qRT-PCR)

Total RNA of tissues and cells was extracted using TRIzol Reagent (Magen) following the manufacturer’s instructions. Reverse transcription was performed using the ABScript III RT Master Mix (ABclonal). The relative expression of each cDNA was determined by quantitative real-time PCR using Universal SYBR Green Fast qPCR Mix (ABclonal). The amplification was carried out for 5 min at 95 °C, followed by 45 cycles of 95 °C for 5 s, 58 °C for 30 s, and 72 °C for 30 s. The analysis of fluorescent signals was performed using a Light Cycler/Light Cycler 480 System (Roche). The viral RNA abundance of SVCV was calculated using standard curves. The relative mRNA levels of different genes were calculated using the 2^−ΔΔthreshold^ cycle method. The primers used in this study can be found in Table S1.

### Viral plaque assay

EPC cells were cultured to confluency in 12-well plates. The virus from cell supernatants was then diluted in a gradient and inoculated onto monolayers of cells. After an incubation period of 1 h, the cells were washed with serum-free DMEM and cultured in DMEM containing 1.5% sodium carboxymethylcellulose (Sigma-Aldrich) and 3% FBS. Visible plaques were counted after 3 days of incubation, and virus titers were calculated.

### Immunoassays

For immunoblot analysis, cells were harvested using cold PBS and then centrifuged at 12,000*g* for 5 min, the precipitates were collected. Immunoprecipitants or whole-cell extracts were separated using 10% SDS-PAGE and transferred onto a polyvinylidene difluoride (PVDF) membrane (Bio-Rad). The membranes were blocked with 2% bovine serum albumin (BSA) for 1 h and subsequently incubated with either rabbit anti-β-actin (dilution of 1:10,000; ABclonal) or anti-SVCV-G monoclonal antibodies [36] for 2 h. Rabbit anti-β-actin antibodies were employed as an internal control. Following washing with TBST, the membranes were incubated with secondary antibodies, either horseradish peroxidase (HRP)-conjugated goat anti-mouse or anti-rabbit antibodies (dilution of 1:2000; ABclonal), for a duration of 45 min. Subsequently, the reactive proteins were detected using a chemical luminescence substrate (General Electric) by the Amersham Imager 600 (General Electric).

### Histopathological examination

Samples of the liver, kidney, and intestine were resected and then fixed with 4% paraformaldehyde (Biosharp). The fixed samples were embedded in paraffin, sectioned, and stained with hematoxylin/eosin (H&E stain). The resulting slices were observed using an Imager microscope (Olympus, Japan).

### Statistical analysis

Statistical analysis was performed using software such as GraphPad Prism. The figure legends provide detailed descriptions of the statistical methods used.

## Results

### Temperature affects the infectivity and pathogenicity of SVCV in zebrafish

**Fig. 1:**
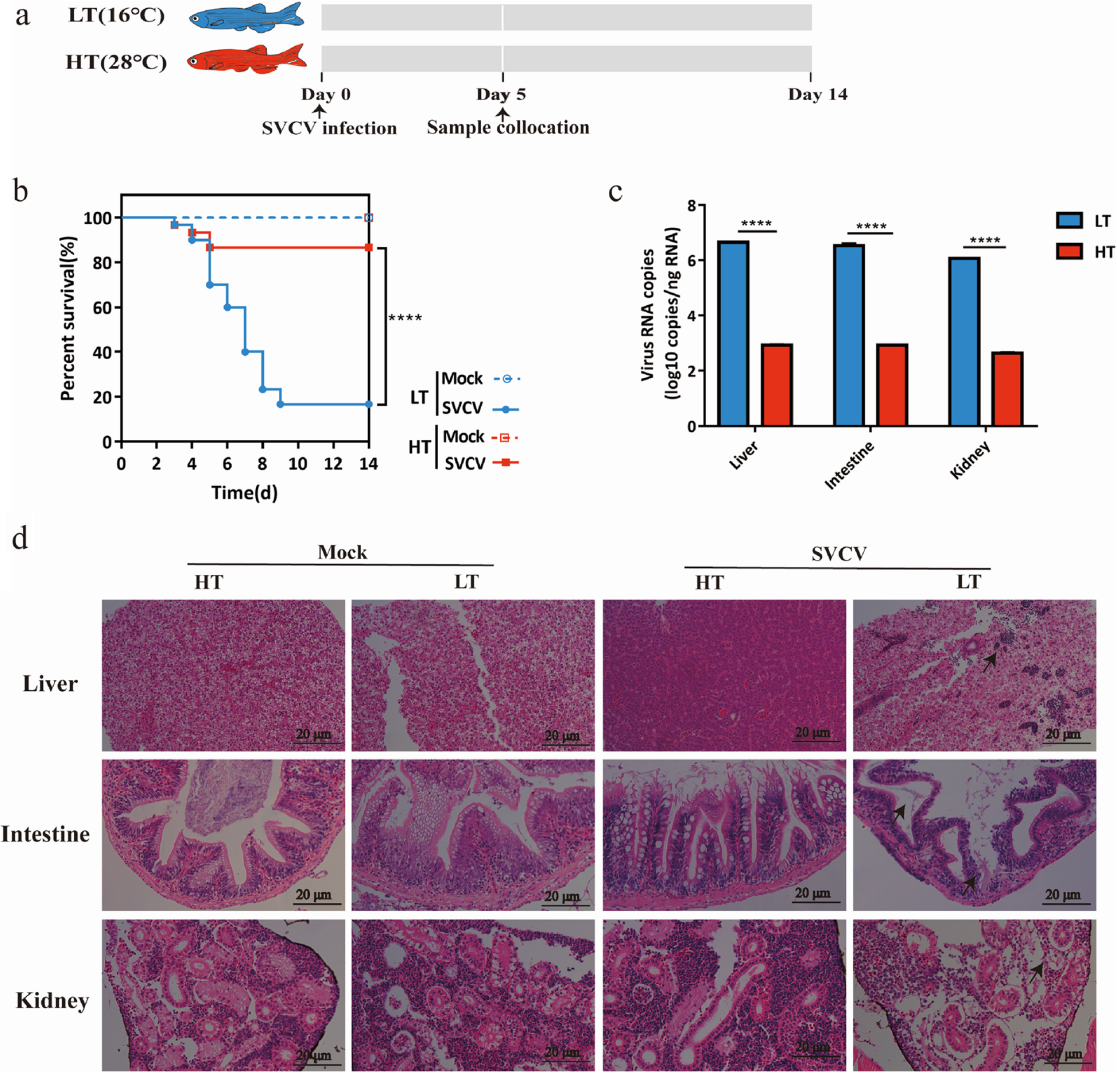
Temperature affects the pathogenicity of SVCV in zebrafish. **a** Schematic of the experiment performed in zebrafish. Zebrafish were infected with SVCV after adapting to the corresponding temperature (16 °C/28 °C), and survival was recorded for 14 days and sampled on the fifth day. **b** The survival rate of zebrafish infected with SVCV at different temperatures (16 °C/28 °C), (*n* = 30 zebrafishes per group). Statistics of survival rate were calculated with the log-rank (Mantel-Cox). ****p < 0.0001. **c** Viral RNA abundance of SVCV at 5 dpi from the liver, intestine, and kidney (*n* = 3 zebrafish per group, 3 technical replicates, mean ± SEM). The statistics were analyzed using two-way ANOVA with Sidak’s post-test. ****p < 0.0001. **d** Histological examination (H&E staining) of the liver, intestine, and kidney at different temperatures after SVCV infection. Representative images from 3 zebrafish are shown. Arrows indicate significant lesions in the liver, intestine, and kidney. Scale bar, 20 μm. All the experiments were independently repeated at least twice, yielding consistent results. The representative data are presented

SVC is known to be more prevalent during spring at water temperatures ranging from 10 to 17 °C, but the disease incidence ceases when water temperature exceeds 22 °C. To verify the temperature-dependent infectivity of SVCV experimentally, the zebrafishes were infected with 10^4^ PFU SVCV at distinct temperatures (16 °C and 28 °C) (Fig. 1a). The results showed that zebrafish are more susceptible to SVCV at low temperature. The survival rate of infected zebrafish at high temperature (83.3%) was significantly higher as compared to the rate at the low-temperature condition (13.3%) (*P* < 0.0001) (Fig. 1b). Additionally, a substantially higher viral load was detected in various tissues of zebrafish in the LT group compared to the HT group (Fig. 1c). Pathological examination of the liver revealed normal hepatocyte morphology and equal cell size after SVCV infection at HT, whereas SVCV infection at LT resulted in structurally incomplete livers infiltrated with inflammatory cells. Similarly, zebrafish infected with SVCV at HT showed intact intestinal structures without noticeable lesions, whereas infection at LT led to incomplete intestinal villi structures and atrophied epithelial cells. Pathological sections of the kidney displayed intact tubular and glomerular structures with tightly arranged cells in the HT group, while the LT group exhibited sparse arrangement of renal cells and loss of renal structures (Fig. 1d). These results indicate that water temperature affects the infectivity and pathogenicity of SVCV in zebrafish.

### Host intestinal microbiota is associated with the infectivity of SVCV at different water temperatures

**Fig. 2:**
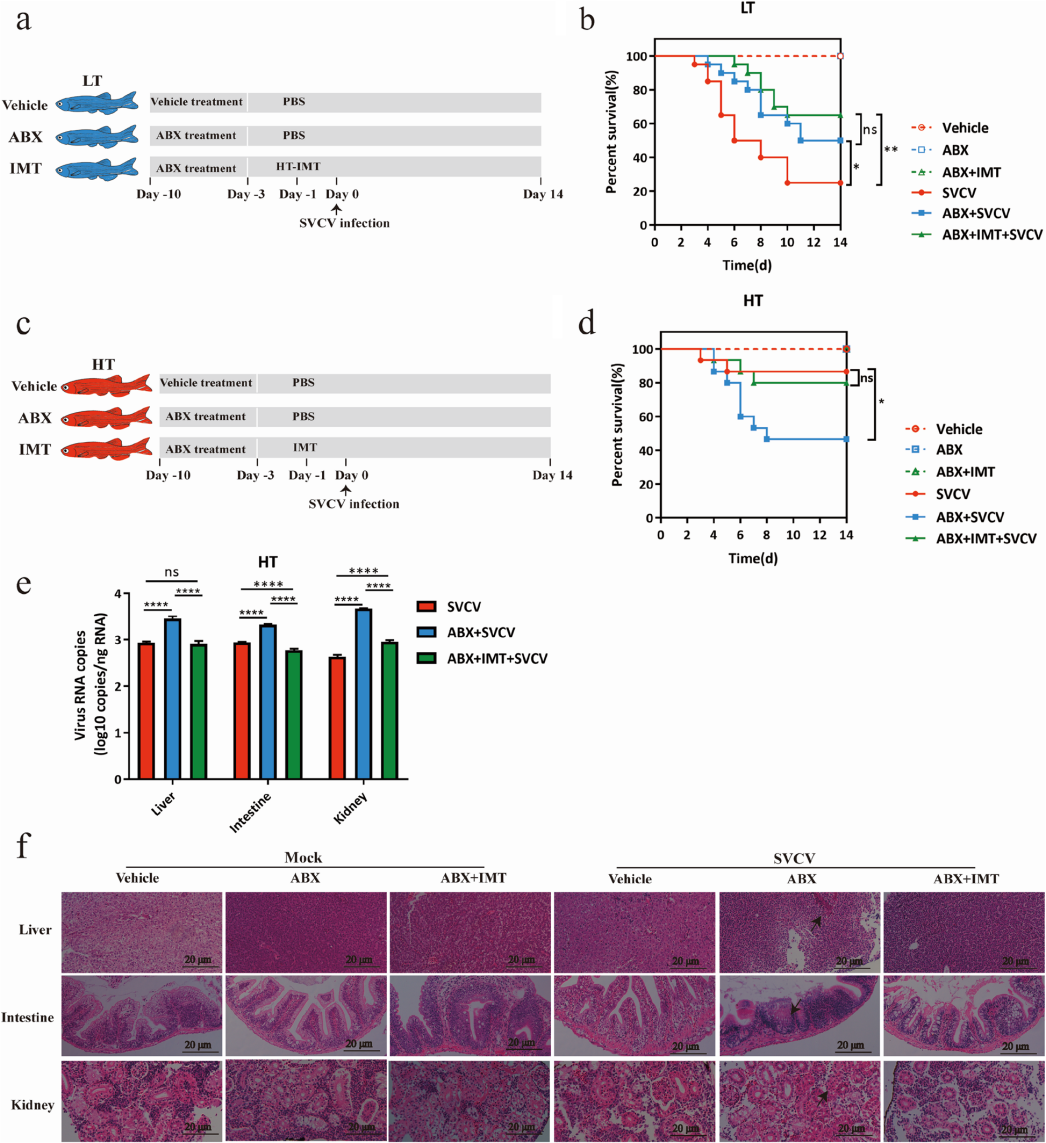
Host intestinal microbiota is associated with the infectivity of SVCV at different temperatures. **a** Schematic of the IMT experiment performed with zebrafish at LT. Zebrafish raised at 16 °C were treated with ABX or vehicle for 7 days, followed by IMT from zebrafish at HT, with PBS as a negative control. They were then infected with SVCV, and their survival was monitored for 14 days. **b** The survival rate of zebrafish in each group (*n* = 20 zebrafish per group). The statistics were analyzed using the log-rank (Mantel-Cox). ns, not significance; *P < 0.05; **P < 0.01 **c** Schematic of the IMT experiment performed with zebrafish at HT. Zebrafish raised at 28 °C were treated with ABX or vehicle for 7 days, followed by IMT from zebrafish at HT, with PBS as a negative control. They were then infected with SVCV, and their survival was monitored for 14 days. **d** The survival rate of zebrafish in each group (*n* = 15 zebrafish per group). The statistics were analyzed using the log-rank (Mantel-Cox). ns, not significance; * *p* < 0.05. **e** Viral RNA abundance of SVCV at 5 dpi from the liver, intestine, and kidney (*n* = 3 zebrafish per group, 3 technical replicates, mean ± SEM). The statistics were analyzed using two-way ANOVA with Sidak’s post-test. ns, not significance; ****p < 0.0001. **f** H&E staining of the liver, intestine, and kidney at 28 °C after SVCV infection. Representative images are shown from 3 zebrafish. Arrows indicate significant lesions in the liver, intestine, and kidney. Scale bar, 20 μm. All the experiments were independently repeated at least twice, yielding consistent results. The representative data are shown

The intestinal microbiota is susceptible to changes in temperatures [14], and different compositions of intestinal microbiota have significant effects on host immunity and viral infections [20]. To investigate the potential relationship between intestinal microbiota and the difference in SVCV infection at varying temperatures, zebrafish were fed with ABX and raised at HT (28 °C) and LT (16 °C). The results showed increased lethality in the HT group, while a decreased lethality was observed in the LT group (Fig. 2b, d), indicating that the intestinal microbiota in zebrafish raised at HT and LT presents different effects on SVCV infection and pathogenicity. The intestinal microbiota of zebrafish at HT may play an anti-viral role, while microbiota in the gut of zebrafish at LT may benefit from SVCV infection.

To validate our speculation, we colonized the zebrafish at LT with the intestinal microbiota from zebrafish raised at HT. Following the process shown in Fig. 2a, zebrafish at LT were treated with ABX and colonized with intestinal microbiota from zebrafish at HT. The survival rate of zebrafish was increased by 25% after ABX treatment upon SVCV infection and further increased by 15% after colonization of intestinal microbial from zebrafish raised at HT (Fig. 2b). These findings indicate that the intestine of zebrafish kept in LT environments may harbor bacteria that are beneficial for SVCV infection, whereas the intestine of zebrafish in HT environments may contain anti-viral bacteria.

To further confirm the presence of SVCV-resistant bacteria in zebrafish intestine at HT, the zebrafish were treated with ABX and housed at HT. Subsequently, they were colonized by the intestinal microbiota of zebrafish at HT followed by SVCV infection (Fig. 2c). ABX treatment in zebrafish raised at HT increased susceptibility to disease, with significantly reduced survival (Fig. 2d), higher viral load (Fig. 2e), and increased liver, intestine, and kidney lesions (Fig. 2f). While after the gut microbes from the HT group were colonized following ABX treatment, the mortality rate, the viral load, and the lesions in the liver, intestine, and kidney of infected fish returned to normal level (Fig. 2d–f). These data reveal that the intestinal microbiota of zebrafish at HT plays a protective role against SVCV infection.

### Comparison of the intestinal microbiota composition of zebrafish at different water temperatures

**Fig. 3:**
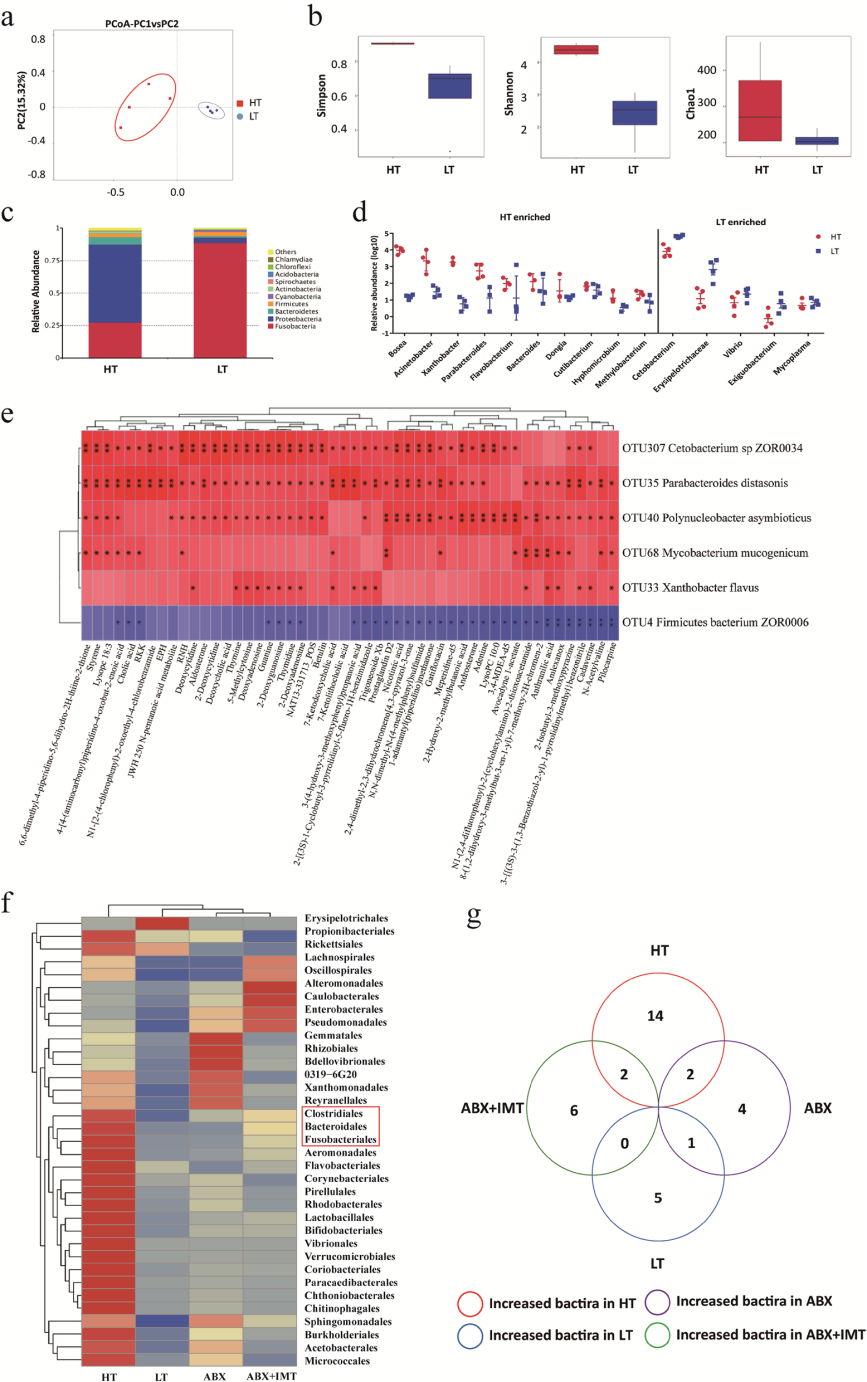
Characterization of the microbial features of zebrafish at different water temperatures. Intestines of zebrafish from HT and LT environments were collected for 16S rRNA sequencing and untargeted metabolomics, with each group consisting of 4 replicates (*n* = 4) and each replicate comprising 3 individuals. The samples were homogenized, total DNA was extracted for sequencing of the 16S rRNA V4 region, and the metabolites were harvested for LC/MS analysis. **a** Samples were clustered into the HT group and LT group by PCoA based on Bray–Curtis distance (*n* = 4). The microbial composition structure differed significantly as analyzed by PerMANOVA (*P* < 0.001), ANOSIM (*P* < 0.001), and MRPP (*P* < 0.001). **b** The α-diversity comparison between the HT group (*n* = 4) and the LT group (*n* = 4). Shannon index, *P* < 0.05; Simpson index, *P* < 0.05; Chao1 index, *P* = 0.223. **c** Relative abundances of bacteria at phyla level in the HT group and LT group. **d** Genera between HT group and LT group are different (*n* = 4). The box plot illustrates that the relative abundance of ten genera was abundant in the HT group and the relative abundance of five genera was enriched in the LT group. **e** The Spearman correlation between intestinal microbiotas and metabolites was analyzed in the HT group vs LT group. In the correlation plot, the red color indicates a positive correlation, while the blue color indicates a negative correlation. The intensity of the color represents the strength of the Spearman correlation. Statistical significance is denoted by *P < 0.05 and **P < 0.01. **f** A heat map displaying the distribution of bacterial genera in intestine samples from LT, HT, ABX, and ABX + LMT groups. The color coding represents the relative abundance of bacteria in the group samples, with the gradient color block indicating the corresponding relationship between the color gradient and the value. **g** Venn diagrams illustrating the overlapping of increased bacterial species in LT, HT, ABX, and ABX + LMT groups. The numbers indicate the counts of bacterial species in the subsets. Metastats analysis was employed to identify microbial species with significant differential abundance

To identify the intestinal microbial species critical for regulating SVCV infection, 16S rRNA gene sequencing was conducted to compare the composition of the intestinal microbiota of zebrafish at 16 °C and 28 °C. Principal coordinate analysis (PCoA) demonstrated marked differences in bacterial communities between the LT and HT groups (Fig. 3a). Test fish in the LT group exhibited significantly lower α-diversity compared to the HT group, as supported by the Shannon index (*P* < 0.05), Simpson index (*P* < 0.05), and Chao1 index (*P* = 0.223) (Fig. 3b). In addition, a detailed analysis at the phylum level unveiled distinct changes in bacterial taxonomic composition in the intestinal microbiota of zebrafish at different temperatures (Fig. 3c). Ten genera including *Bosea*, *Acinetobacter*, *Xanthobacter*, *Parabacteroides*, *Flavobacterium*, *Dongia*, and *Bacteroides* were all enriched in the HT group, whereas five genera, including *Cetobacterium*, *Erysipelotrichaceae*, *Vibrio*, *Exiguobacterium*, and *Mycoplasma*, were more abundant in the LT group (Fig. 3d). Bacteria belonging to these genera, exhibiting notable variations at high and low temperatures, could potentially impact the infectivity of SVCV under different water temperature conditions.

To investigate the relationship between intestinal microbiota, metabolites, and SVCV infection at the selected temperatures, we explored metabolic profiling using high-throughput LC/MS. PCA based on metabolic revealed striking differences in microbial functional structures between the LT and HT groups (Fig. S1a). A total of 417 compounds from the KEGG compound database were identified (Fig. S1b-c). After KEGG annotation, 57 compounds, including DCA, 7-ketoLCA, 7-ketoDCA, and avocadyne 1-acetate, were enriched in the HT group, and 116 compounds such as GlcADG, LPS, stachyose, and verbascose were enriched in the LT group. Associations were observed between intestinal microbiota and their mediated metabolites, including nucleic acids, organic acids, and lipids. Spearman’s correlation analysis revealed positive associations between bacteria such as *Cetobacterium sp ZOR0034*, *Parabacteroides distasonis*, *Polynucleobacter asymbioticus*, and *Mycobacterium mucogenicum*, and metabolites like nucleic acids, bile acids, and certain lipid metabolites. Conversely, *Firmicutes bacterium ZOR0006* displayed a negative correlation with pilocarpine, cadaverine, and anthranilic acid (Fig. 3e). These bacteria show a strong correlation with metabolites in the intestine of affected zebrafish and thus may be linked to altered infectivity of SVCV at different water temperatures.

To further identify the specific bacteria responsible for the primary antiviral effect at HT, we conducted 16S rRNA gene sequencing on the intestinal microbiota of zebrafish after treating them with ABX or colonizing them with intestinal microbiota following ABX treatment (ABX + IMT) at HT for comparison. The cluster analysis revealed that the abundance of *Bacteroidales*, *Fusobacteriales*, and *Clostridiales* decreased significantly in ABX-treated zebrafish while increased in microbiota-transplanted zebrafish as shown in heatmaps (Fig. 3f). Further analysis combining the microbiome data from zebrafish at different temperatures and treated with ABX or ABX + IMT revealed that *Parabacteroides distasonis* and *Cetobacterium somerae* were consistently found in both HT and ABX + IMT groups, but rarely in the LT and ABX groups. On the other hand, *Bosea vestrisii* and *Xanthobacteraceae* showed higher abundance in the HT and ABX groups, but lower abundance in the LT and ABX + IMT groups. Additionally, *Firmicutes bacterium ZOR0006* exhibited increased abundance in the LT and ABX groups, while showing decreased abundance in the HT and ABX + IMT groups (Fig. 3g). These findings demonstrated the likely correlation of *Parabacteroides distasonis* and *Cetobacterium somerae* with the infectivity of SVCV at different water temperatures.

### *Parabacteroides distasonis* restricts SVCV infection in zebrafish

**Fig. 4:**
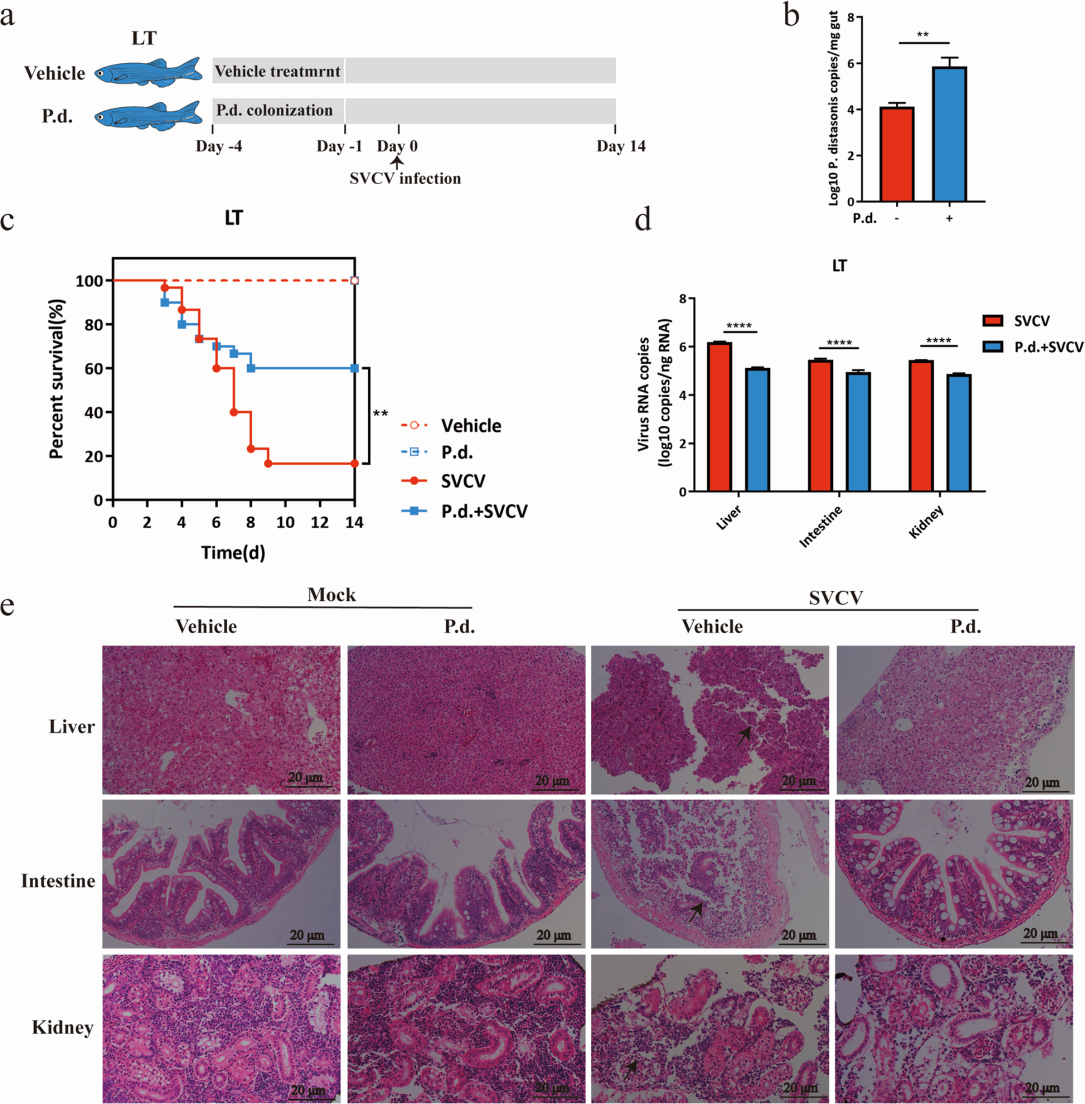
Colonization with *Parabacteroides distasonis* at LT restricts SVCV infection. Schematic of *Parabacteroides distasonis* colonization in zebrafish at LT. Zebrafishes housed at 16 °C were colonized with *Parabacteroides distasonis* or treated with vehicle as negative control, followed by infection with SVCV. The survival of zebrafish was recorded for 14 days. **b** The abundance of *Parabacteroides distasonis* in the intestinal microbiota of zebrafish after colonization (*n* = 3 zebrafish per group, 3 technical replicates, mean ± SEM). Student’s *t*-test. ** *P* < 0.01. **c** The survival rate of zebrafish in each group is a control (*n* = 30 zebrafish per group). The statistics were analyzed using the log-rank (Mantel-Cox). ** *P* < 0.01. **d** Viral RNA abundance of SVCV at 5 dpi from the liver, intestine, and kidney (*n* = 3 zebrafish per group, 3 technical replicates, mean ± SEM). The statistics were analyzed using two-way ANOVA with Sidak’s post-test. ****P < 0.0001. **e** H&E staining of the liver, intestine, and kidney at 16 °C after SVCV infection. Representative images are shown from three zebrafish. Arrows indicate significant lesions in the liver, intestine, and kidney. Scale bar, 20 μm. All experiments were independently repeated at least twice, yielding similar results. The representative data are presented

In light of the higher abundance of *Parabacteroides distasonis* compared to *Cetobacterium somerae* in zebrafish raised at high temperature, as well as the observed correlation between *Parabacteroides distasonis* and higher levels of metabolites in zebrafish at HT, we further validated its role in regulating the infectivity of SVCV based on temperature. Zebrafish raised at LT were orally administrated with *Parabacteroides distasonis* prior to SVCV infection (Fig. 4a). The qPCR assay confirmed successful colonization of *Parabacteroides distasonis* in the intestine of zebrafish (Fig. 4b). Remarkably, colonization with *Parabacteroides distasonis* significantly increased the survival of zebrafish infected with SVCV at LT (*P* = 0.0054) (Fig. 4c). Additionally, a significant reduction of viral load across all organs and diminished organ lesions were observed following *Parabacteroides distasonis* colonization (Fig. 4d, e). These results suggest that *Parabacteroides distasonis* plays a critical role in preventing SVCV infection.

### Production of bile acid is associated with the infectivity of SVCV at different water temperatures

**Fig. 5:**
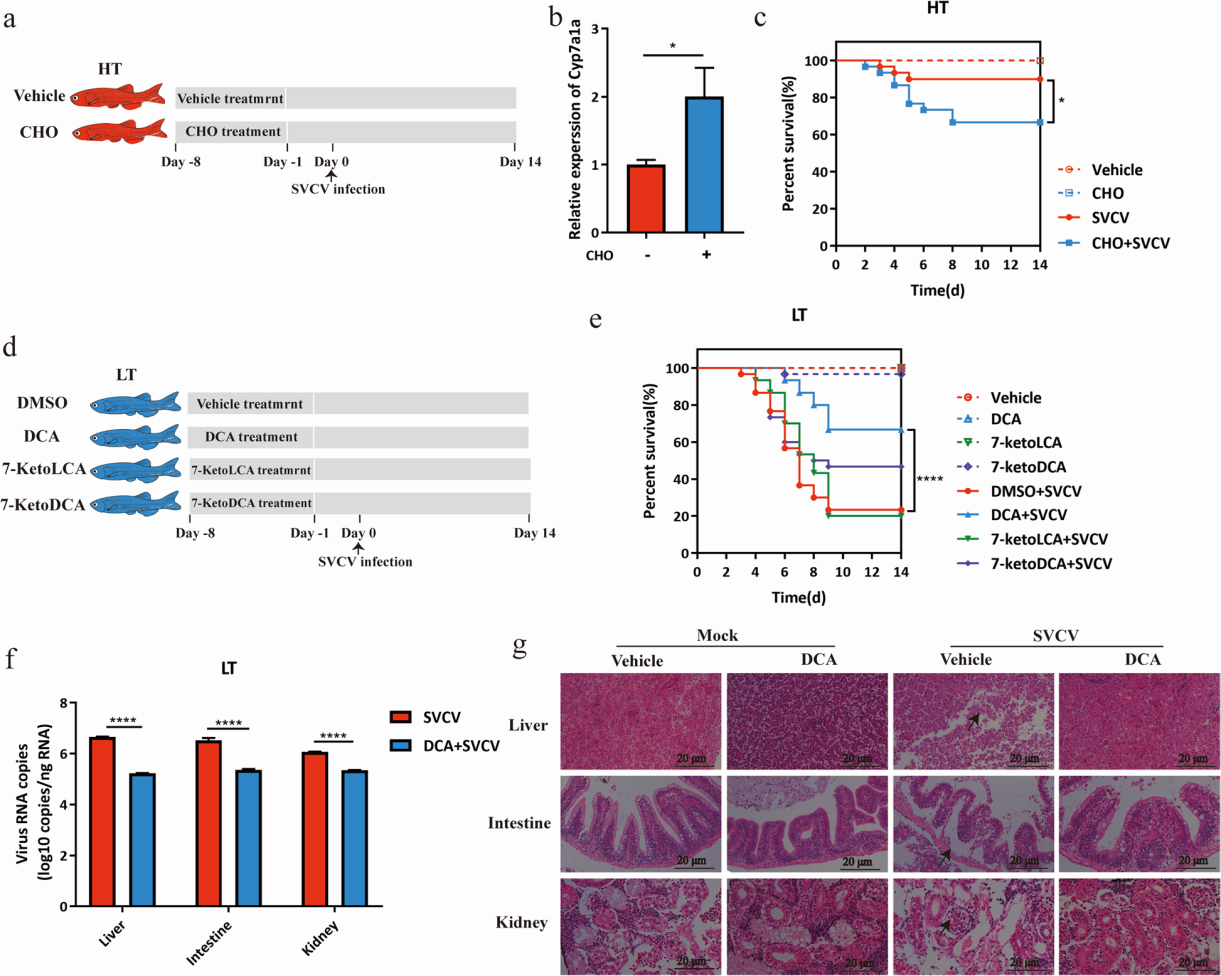
Production of bile acid is associated with the infectivity of SVCV at different temperatures. **a** Schematic of the CHO treatment experiment. Zebrafish were fed with CHO or vehicle, followed by SVCV infection. The survival of zebrafish was recorded for 14 days. **b** The relative expression of *Cyp7a1a* in the liver of zebrafish after CHO treatment (*n* = 3 zebrafish per group, 3 technical replicates, mean ± SEM). Student’s *t*-test. *P < 0.05. **c** The survival rate of zebrafish in each group (*n* = 30 zebrafish per group). The statistics were analyzed using the log-rank (Mantel-Cox). *P < 0.05. **d** Schematic of bile acids administration assay. Zebrafish raised at 16 °C were treated with 1mM of DCA, 7-ketoLCA, or 7-ketoDCA diluted in DMSO or an equal volume of DMSO, followed by infection with SVCV, and the survival of zebrafish was recorded for 14 days. **e** The survival rate of zebrafish in each group (*n* = 30 zebrafish per group). The statistics were analyzed using the log-rank (Mantel-Cox). **** *p* < 0.0001. **f** Viral RNA abundance of SVCV at 5 dpi from the liver, intestine, and kidney after treatment (*n* = 3 zebrafish per group, 3 technical replicates, mean ± SEM). The statistics were analyzed using two-way ANOVA with Sidak’s post-test. ****p < 0.0001. **g** H&E staining of the liver, intestine, and kidney at 16 °C after SVCV infection. Representative images are shown from 3 zebrafish. Arrows indicate significant lesions in the liver, intestine, and kidney. Scale bar, 20 μm. All experiments were independently repeated at least twice, yielding similar results. The representative data are shown

Considering the significant role of *Parabacteroides distasonis* in the conversion of the primary bile acids to various secondary bile acids, including DCA, ursodeoxycholic acid (UDCA), lithocholic acid (LCA), and a strong association observed between multiple bile acids and *Parabacteroides distasonis* in our study (Fig. 3e), we next investigated the effect of bile acids on SVCV infection. CHO was added to the diet of zebrafish raised at HT to induce the sequester of bile acid (Fig. 5a). A significant upregulation of the expression of *cyp7a1a*, the bile acid synthesis gene, indicating that bile acid level was successfully reduced by cholestyramine treatment (Fig. 5b). Following infection of zebrafish with SVCV, we observed a significant decrease (23.3%) in their survival rates after administering cholestyramine (Fig. 5c), suggesting the inhibitory effect of bile acid on SVCV infection. To further explore the role of secondary bile acids on SVCV infection, three specific secondary bile acids (DCA, 7-ketoLCA, and 7-ketoDCA) that are closely associated with *Parabacteroides distasonis* were orally administered to zebrafish cultured at LT followed by SVCV infection (Fig. 5d). We found that administration of DCA resulted in a significant increase (43.4%) in the survival rate of the recipient zebrafish with SVCV infection (*P* < 0.0001) (Fig. 5e). In addition, oral administration of DCA to zebrafish at LT prior to SVCV infection resulted in reduced viral load in the liver, intestine, and kidney (Fig. 5f) and significantly decreased organ lesions (Fig. 5g). These findings reveal that the production of secondary bile acid, DCA, mediated by *Parabacteroides distasonis* contributes to the anti-SVCV effect.

### DCA inhibits SVCV assembly and release in EPC cells

**Fig. 6:**
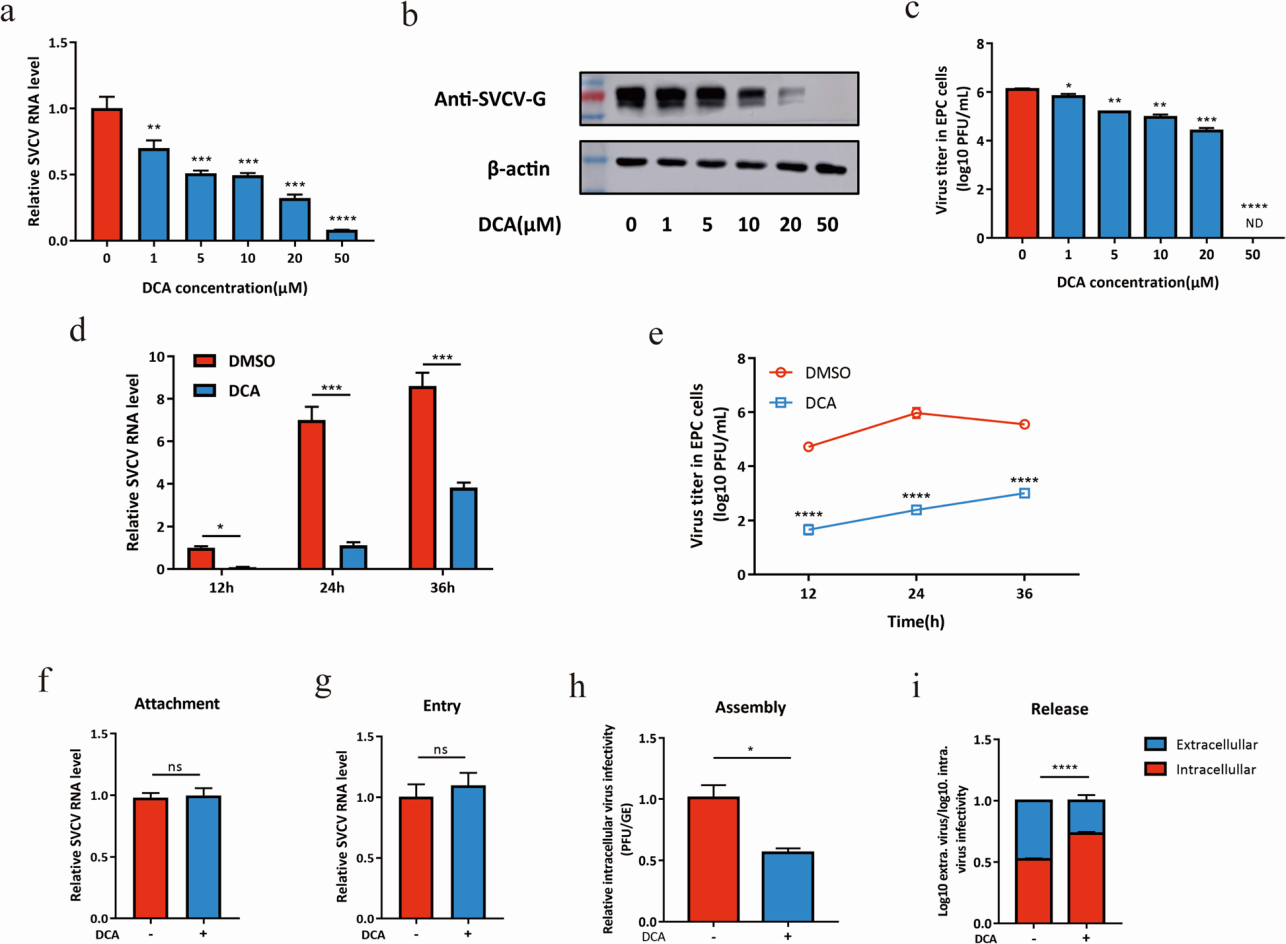
DCA inhibits SVCV assembly and release. **a**–**c** EPC cells were pretreated with DCA (0–50 μM) for 12 h and then infected with SVCV at an MOI of 0.1. Cell samples and supernatants were collected at 24 hpi, and the viral RNA and protein levels and infectious virus titers were then determined by qRT-PCR (**a**), Western blot (**b**), and plaque formation assay (**c**), respectively. The statistics were analyzed using one-way ANOVA with Sidak’s post-test. **P < 0.01, ***P < 0.001, ****P < 0.0001, ND, no detection. **d**, **e** EPC cells were pretreated with 50 μM of DCA for 12 h and then infected with SVCV at an MOI of 0.1. Cell samples and supernatants were collected at the indicated time points, and the viral RNA levels and infectious virus titers were measured by qRT-PCR (**d**) and plaque formation assay (**e**), respectively. The statistics were analyzed using two-way ANOVA with Sidak’s post-test. *P < 0.05, ***P < 0.001, ****P < 0.0001. **f** EPC cells were incubated with SVCV at an MOI of 10 in the absence or presence of DCA (20 μM) at 4 °C for 1 h. Subsequently, the cells were washed three times with ice-cold PBS, and the RNA abundance of the attached virus was evaluated using qRT-PCR. The significant difference was analyzed by Student’s *t*-test. ns, no significance. **g** After incubation with SVCV (MOI = 10) at 4 °C for 1 h, the EPC cells were then shifted to 28 °C in the absence or presence of DCA (20 μM) for 1 h. Subsequently, the cells were washed three times with ice-cold PBS, and the intracellular viral RNA was quantified by qRT-PCR. The significant difference was analyzed by Student’s *t*-test. ns, no significance. **h** EPC cells were pretreated with DCA (20 μM) for 12 h, followed by SVCV infection (MOI = 10). The efficiency of the viral assembly was assessed by comparing the infectious virus titers with the total SVCV genome equivalents (GE) at 12h post-infection. The statistics were analyzed by Student’s *t*-test. *P < 0.05. **i** EPC cells were pretreated with DCA (20 μM) for 12 h, followed by SVCV infection (MOI = 10). The efficiency of virus release was assessed as the ratio of intracellular and extracellular infectivity relative to the total infectivity at 12 h post-infection. The statistics were analyzed using two-way ANOVA with Sidak’s post-test. ***P < 0.001. All data shown were pooled from 2 independent experiments performed in triplicate (*n* = 6, mean ± SD)

To investigate the inhibitory mechanism of DCA on SVCV infection, an increasing amount of DCA which showed no cellular toxicity was added to EPC cells followed by SVCV infection (Fig. S2a). The abundances of viral RNA and protein and infectious viral titers were measured by qRT-PCR, western blot, and plaque assay, respectively, at 24 hpi. The qRT-PCR analysis revealed a downregulation of SVCV RNA in DCA-treated EPC cells (Fig. 6a). Similarly, the expression of SVCV G protein was also reduced upon DCA treatment (Fig. 6b). Moreover, viral plaque assay showed a decrease in the production of infectious SVCV progeny in response to DCA treatment (Fig. 6c), indicating the concentration-dependent antiviral activity of DCA against SVCV. To further verify this result, EPC cells were treated with 50 μM DCA prior to SVCV infection, and the cells were collected at 12, 24, and 36 hpi. As expected, DCA treatment was found to reduce viral replication at different time points after SVCV infection (Fig. 6d, e).

To determine the impact of DCA on viral proliferation at various stages of the SVCV life cycle, the viruses were incubated with DCA at 4 °C for 1 h. The attachment of viruses to cells was then assessed by qRT-PCR analysis. The results showed comparable levels of viral RNA on EPC cells with or without DCA treatment, indicating that DCA did not affect viral attachment (Fig. 6f). Next, we examined the influence of DCA on viral entry. EPC cells were incubated with SVCV at 4 °C for 1 h to allow attachment, followed by incubation in the absence or presence of DCA for 2 h at 28 °C to facilitate viral entry. Quantification of invading SVCV using qRT-PCR analysis of the viral genome demonstrated that DCA did not interfere with the viral entry (Fig. 6g). Furthermore, we evaluated the effect of DCA on the assembly and release of SVCV at 12 hpi which is the time of the completion of the viral replication cycle. The results showed that the efficiency of SVCV assembly decreased in response to DCA treatment, as determined by comparing the ratio of infectious virus to viral RNA (Fig. 6h). Moreover, DCA treatment reduced the ratio of the titers of extracellular SVCV to intracellular SVCV, suggesting a potential impact on viral release (Fig. 6i).

### DCA inhibits the replication and pathogenicity of IHNV both in vivo and in vitro

**Fig. 7:**
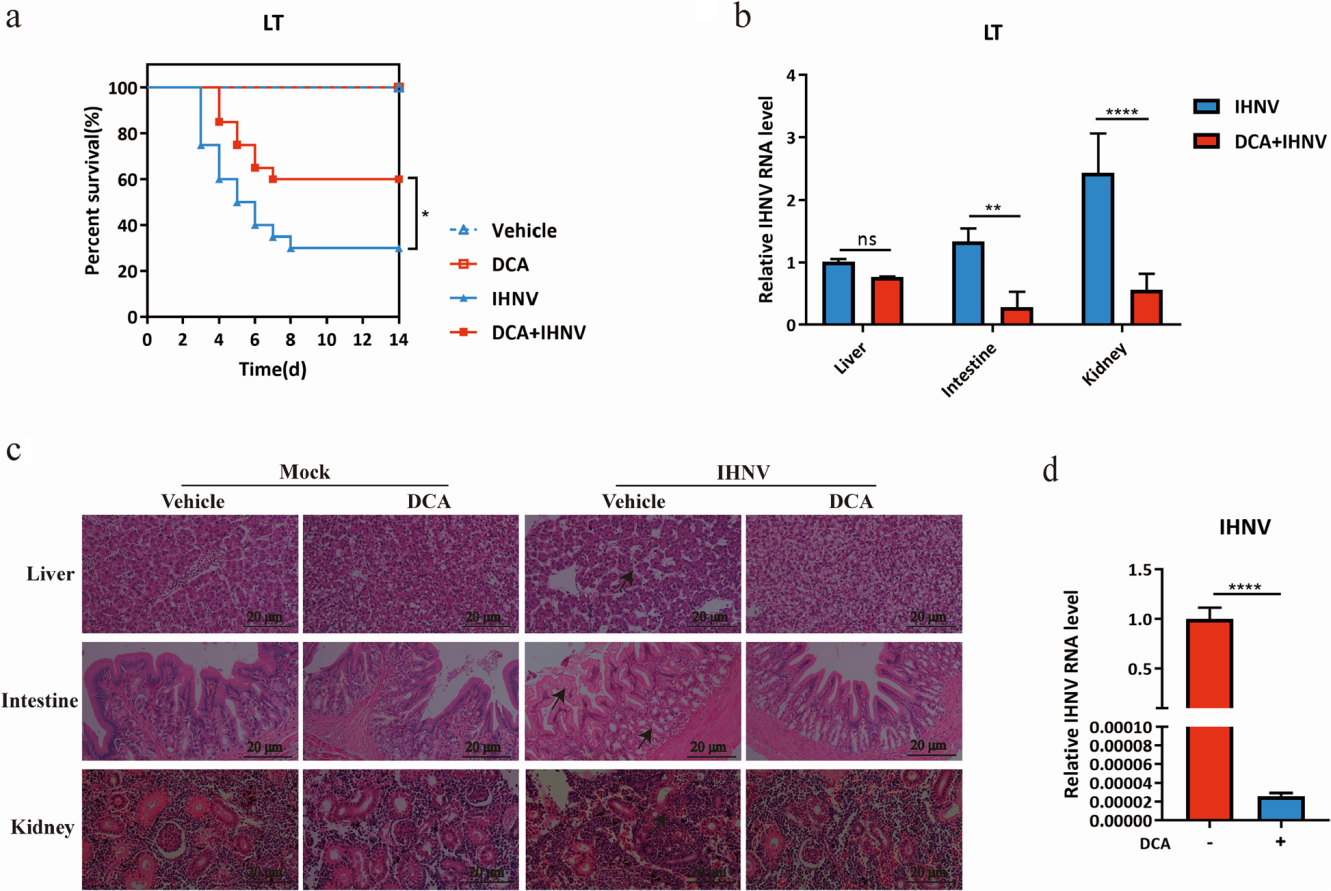
DCA inhibited IHNV infection in vivo and in vitro. **a**–**c** Rainbow trout were administrated with DCA or vehicle for 7 days, followed by IHNV or mock infection at 16 °C, and the survival of rainbow trout was recorded for 14 days. **a** The survival rate of rainbow trout in each group was shown (*n* = 30 rainbow trout per group). The statistics were analyzed using the log-rank (Mantel-Cox). * *P* < 0.05. **b** Viral RNA abundance of IHNV at 5 dpi from the liver, intestine, and kidney after DCA administration (*n* = 3 rainbow trout per group, 3 technical replicates, mean ± SEM). The statistics were analyzed using two-way ANOVA with Sidak’s post-test. **P < 0.01, ****P < 0.0001, ns, no significance. **c** H&E staining of the liver, intestine, and kidney at 16 °C after IHNV infection. Representative images are shown from 3 rainbow trout. Arrows indicate significant lesions in the liver, intestine, and kidney. Scale bar, 20 μm. All experiments were independently repeated at twice, yielding similar results. The representative data are shown. **d** EPC cells were pretreated with 50 μM of DCA for 12 h, followed by IHNV infection (MOI = 5). The RNA levels of IHNV were assessed by qRT-PCR. Data presented were pooled from two independent experiments performed in triplicate (*n* = 6, mean ± SD). The statistics were analyzed by Student’s *t*-test. **** *P* < 0.0001

To explore the effect of DCA on other viruses in the family *Rhabdoviridae* showing high infectivity at LT, we incorporated DCA into the feed of rainbow trout cultured at 16 °C for 7 days. Subsequently, the rainbow trout were injected with 10^4^ PFU IHNV. The result revealed that treatment with DCA resulted in a 30% increase in the survival rate of rainbow trout after IHNV infection (Fig. 7a), and substantially reduced viral load in the kidney and intestine (Fig. 7b). Histopathological analysis revealed that DCA supplementation mitigated the pathological damage caused by IHNV infection in the liver, kidney, and intestine (Fig. 7c). To validate the effect of DCA on IHNV replication, the in vitro experiments were carried out and showed that pretreatment of EPC cells with 50 μM of DCA significantly diminished the viral load following IHNV infection (Fig. 7d). These findings clearly show that DCA may have broad effects on viruses in family *Rhabdoviridae* which are known to be highly infectious to fish at LT.

## Discussion

Temperature-dependent infection is a critical characteristic of pathogenic viruses, which is derived through co-evolution with the host and adaptation to the environment [37, 38]. In this study, we investigated the impact of temperature on the susceptibility of zebrafish to SVCV infection. Our results demonstrated that the composition of intestinal microbiota is associated with temperature-based SVCV sensitivity. *Parabacteroides distasonis* and its metabolite DCA were identified as critical factors for inhibiting SVCV infection at high temperature. Furthermore, DCA also significantly inhibits other viruses in the family *Rhabdoviridae* that are more infectious at low temperature. These findings may form an essential basis for the development of more effective approaches for preventing and controlling SVCV infection and for studying other viruses that cause diseases at specific temperature ranges.

Mammals are homotherms with a limited change of body temperature, which leads to a narrower range of temperature for viral infection. This has been observed in various mammalian viruses, such as rhinovirus, flavivirus, influenza virus, and SARS-CoV-2 [37]. The mechanisms affecting the temperature-based sensitivity of these viruses have been linked to host immunity [39, 40], virus-host binding activity [41], and virus replication capacity [42]. SARS-CoV-2 can cause significant infections at 33 °C with a slightly mortal rate and symptoms at a higher temperature [43, 44]. This phenomenon is related to the temperature-dependent binding affinity of SARS-CoV-2 to human ACE2 receptor [45]. Rhinovirus, a respiratory virus, replicates optimally at cool temperatures in the nasal cavity (33–35 °C), since human epithelial cells stimulate a more powerful innate immune response [46] and interferon-independent host defense including apoptosis and antiviral ribonuclease, RNAseL at warm temperature [47]. The intestinal microbiota is known to be highly sensitive to environmental temperature, including its composition and abundance [21, 48–50]. Most teleost fishes are poikilotherms, and their body temperature cannot be stably maintained, which results in more variable intestinal microbiota [51–53]. Therefore, it was hypothesized that the intestinal microbiota may regulate the infection of aquatic viruses at different temperatures. To verify this hypothesis, we conducted antibiotic treatment experiments on zebrafish at distinct temperatures and demonstrated that antibiotic treatment reduced the survival of zebrafish infected with SVCV at 28 °C but enhanced zebrafish survival at 16 °C. When colonization with intestinal microbiota, survival rates of zebrafish at high temperature were increased, confirming that zebrafish raised at high temperature contain bacteria that possess anti-SVCV effect in their intestine.

In previous studies, temperature changes were shown to affect intestinal microbial diversity and significantly alter the metabolite profile of the intestinal microbiota of affected fish [26, 54]. Our findings further validate the impact of temperature changes on intestinal microbiota and highlight its significance in viral infections. The intestinal microbiota is known to play a vital role in enhancing the resistance of fish to SVCV. Conventional zebrafish (*Danio rerio*) was found to exhibit greater resistance to SVCV infection compared to germ-free zebrafish [55]. Further investigation can be conducted on the impact of intestinal microbiota on the infectivity of SVCV at different temperatures by utilizing germ-free zebrafish. Subsequently, we developed an effective standardized approach for the effective identification of specific gut microbes that affect temperature-based viral sensitivity in this study. We identified *Parabacteroides distasonis* and *Cetobacterium somerae* two important bacterial species, whose abundance increased in the HT and ABX + IMT groups but decreased in the LT and ABX groups. While other bacteria may be more abundant than *Parabateroides* in HT zebrafish compared to LT zebrafish, they did not show higher abundance in the ABX + IMT group than in the ABX group. This indicates that these bacteria may not play a significant role in regulating SVCV infection at different temperature. Xie et al. previously reported the protective effect of *C. somerae* against SVCV infection in zebrafish [56], providing supportive evidence for our findings. In addition, it is acknowledged that certain bacteria in *Proteobacteria* phylum can promote lethality and other pathologies. Therefore, the high abundance of *Proteobacteria* in zebrafish at HT, along with the high ratio of *Proteobacteria* to *Firmicutes*/*Fusobacteria*/*Bacteroidetes*, may indicate a potential risk to gut health [57]. Further analysis revealed a particularly heightened presence *Bosea* and *Acinetobacter* in HT zebrafish, both recognized as opportunistic pathogens [58, 59]. Some species of these bacteria process immunoactivation properties, the implications of which remain not extensively studied in the context of viral infection [60]. Despite this, given that SVCV is a hemorrhagic virus with high lethality, the adverse effects on the host when SVCV infects zebrafish at LT appear to outweigh the negative impact of *Proteobacteria* on zebrafish at HT.

Our findings have revealed that *Parabacteroides distasonis* and *Cetobacterium somerae* could potentially regulate the SVCV infection at different temperatures. Considering *Parabacteroides distasonis* is more abundant in the HT zebrafish and the positive correlation between *Parabacteroides distasonis* with secondary bile acids, it may suggest their more important role in SVCV infection. *Parabacteroides distasonis* is a Gram-negative probiotic and is known to have beneficial effects on weight gain [61], hyperglycemia and hyperlipidemia [62], hepatic steatosis [63], and reduction of rectal cancer [64]. Previous reports indicated that oral administration of *Parabacteroides distasonis* reduced the level of inflammation in mice, which in turn inhibited the development of colitis and colon cancer [65, 66]. Current studies on *Parabacteroides distasonis* have mainly focused on its function in metabolic and immune regulation, but no report for its impact on viral infection. Our study found that colonization of *Parabacteroides distasonis* can provide an antiviral effect on SVCV in zebrafish. *Parabacteroides distasonis* could use the host primary bile acid to produce secondary bile acids DCA, LCA, and UDCA [67]. There is a strong association between *Parabacteroides distasonis* and several bile acids in the intestine including various secondary bile acids such as DCA, 7-ketoLCA, and 7-ketoLCA. After treating zebrafish raised high temperature with cholestyramine to inhibit the normal function of bile acids [68], high-temperature zebrafish became susceptible to SVCV infection. All these findings indicate that bile acids play an important role in reducing the susceptibility of zebrafish to SVCV infection.

Bile acids are known to modulate the infection of many other viruses such as enterovirus [69, 70], influenza A virus [71], mouse cytomegalovirus [72], chikungunya virus [73], SARS-CoV-2 [74], and hepatitis D virus [75]. The present study demonstrates among various bile acids, DCA is the most effective one in improving the survival of zebrafish after SVCV infection. Other bile acids, such as GCDCA, could enhance the binding of MNOV to the receptor CD300lf to promote cellular adsorption [76]. In addition, GCDCA is also critical for porcine enteric calicivirus to escape from the endosomes into the cell cytoplasm [77]. Cholic acid induces multiple cellular responses, such as rapid changes in caveolae-mediated endocytosis, endosomal acidification, and dynamics of the endosomal/lysosomal system to enhance SADS-CoV replication [78]. In this study, DCA inhibits the assembly and release of SVCV. The use of DCA in vivo and in vitro tests decreased cholesterol content [79, 80], and this component of lipids could also affect the structure of lipid droplets and lipid rafts and thus the assembly and release stages of different viruses [81, 82]. However, it is unclear whether DCA can inhibit SVCV by decreasing cholesterol content. In 2021, Kong et al. demonstrated that local BAs in the liver and intestines, systemic BAs in blood, and different types of BAs played a complex role in the life cycle of different viruses [69]. Many bacteria can also be transformed to produce DCA [83], of which *Clostridium scindens* and its metabolite secondary bile acids (DCA) could affect IFN production in pDCs and thus ISG expression in monocytes, ultimately inhibiting the Chikungunya virus infection [73]. Except for *Parabacteroides distasonis*, none of the bacteria that show significant differences in abundance in the zebrafish intestine at different water temperatures are capable of producing DCA. Further investigation is required to determine whether *Parabacteroides distasonis*’s inhibitory effect on SVCV is solely due to the production of DCA. This can be accomplished by studying *Parabacteroides distasonis* mutants that lack the ability to transform BAs, which would confirm the role of secondary BAs in regulating immune responses against SVCV infection in a *Parabacteroides distasonis*-dependent manner.

Temperature plays a significant role in the susceptibility of the host and infectivity of pathogenic viruses in aquaculture [1]. For example, CyHV-3 [3], GCRV [84], ISKNV [85], NNV [86], etc., are more infectious at high temperatures, while low temperatures make fish more susceptible to HIRRV [4], SVCV [11], IPNV [87], etc. This study has demonstrated the potential broad-spectrum effectiveness of DCA against SVCV and IHNV that cause fish diseases at low temperature, as DCA treatment also significantly prevented the rainbow trout from infection with IHNV. However, further investigation is warranted to understand the underlying reasons and mechanisms that may modulate the infection of these viruses and whether similar mechanisms are involved. This finding may offer a new strategy for controlling and preventing viruses in *Rhabdoviridae* family, particularly those that pose a greater threat to aquatic organisms in colder environments.

## Conclusion

**Fig. 8:**
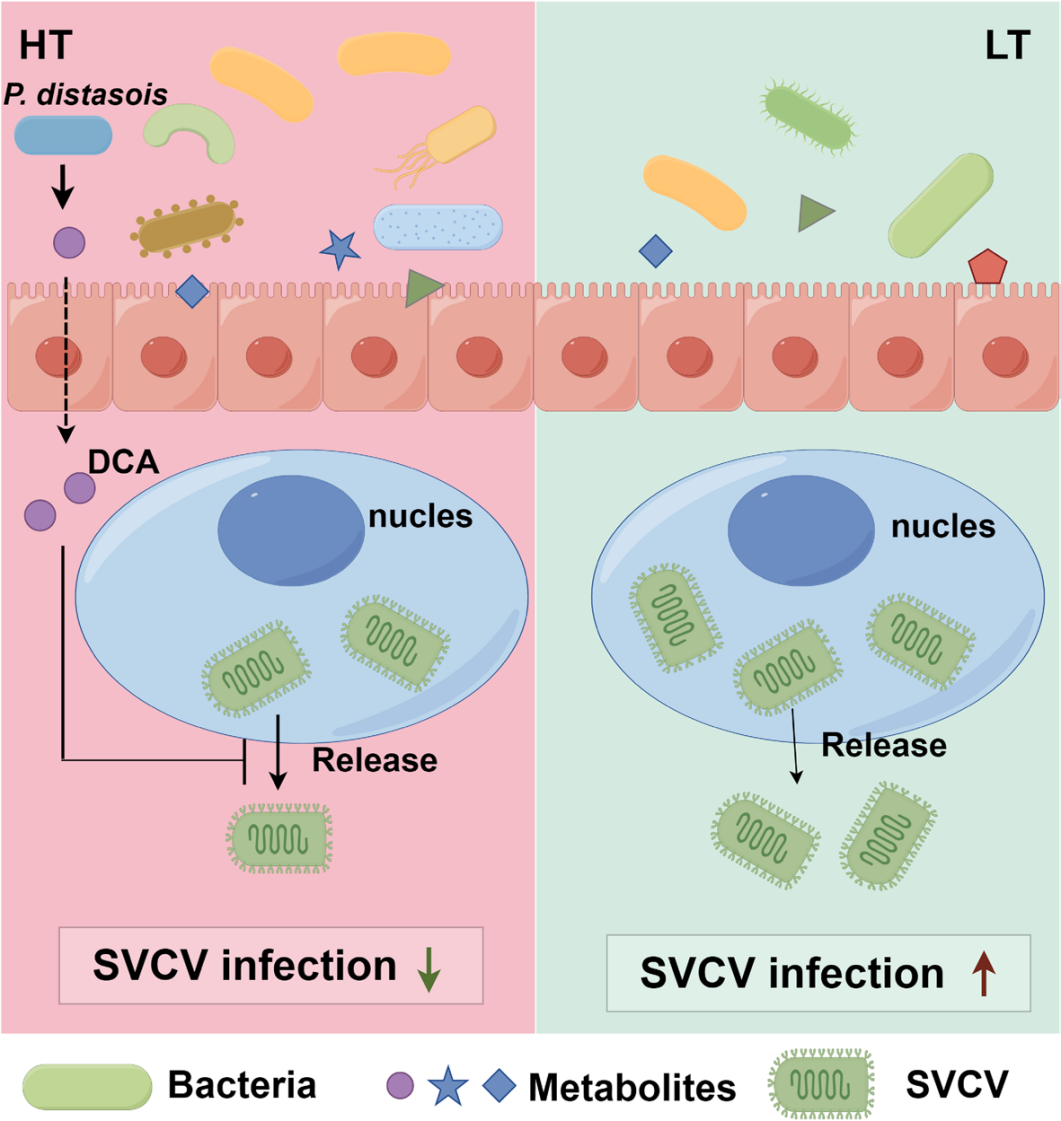
The schematic model illustrating the intestinal microbiota regulates the temperature sensitivity of SVCV. Low temperature (16 °C) is associated with decreased abundance and diversity of intestinal microbiota, creating favorable conditions for significant SVCV infection and subsequent organismal damage. Conversely, high temperature (28 °C) promotes a higher abundance and diversity of zebrafish intestinal microbiota, which limits SVCV infection and minimizes the damage. Additionally, we identify a regulatory role of the DCA, a metabolite of *Parabacteroides distasonis*, in inhibiting SVCV assembly, release, and eventual infection at elevated temperatures

In this study, we confirmed that the infectibility of SVCV is closely related to environmental temperature. The intestinal microbiota which is sensitive to temperature impacted the pathogenicity and infectivity of SVCV. As illustrated in Fig. 8, zebrafish raised at HT exhibited elevated abundance and diversity of intestinal microbiota compared to those at LT. In addition, *Parabacteroides distasonis* is determined to be a highly enriched species in the intestine of zebrafish raised at HT and its production of secondary bile acids DCA inhibited the assembly and release of SVCV. These inhibitory effects of DCA were also observed on IHNV, another *Rhabdoviridae* member that causes diseases at LT. Taken together, our findings from this study will provide new opportunities for exploring how water temperature affects the infectivity and pathogenicity of aquatic viruses and also form a scientific basis for controlling and preventing temperature-sensitive viruses in aquaculture.

## Supplementary Information

**Additional file 1.** Supplementary Figures S1-S2.

**Additional file 2. **Supplementary Tables S1.

## Data Availability

The 16S rRNA gene sequencing data used in this study are available in the NCBI Short Read Archive under Bioproject PRJNA989424 (https://www.ncbi.nlm.nih.gov/bioproject/PRJNA989424). The raw data of metabolomics used in this study are available in the Mendeley Data under DOI 10.17632/2vjvs7fgzc.1 (https://data.mendeley.com/datasets/2vjvs7fgzc/1).

## Abbreviations

SVCV: Spring viremia of carp virus
DCA: Deoxycholic acid
IHNV: Infectious hematopoietic necrosis virus
CyHV-3: Cyprinid herpesvirus 3
HIRRV: Hirame rhabdovirus virus
ABX: Antibiotics
IM: Intestinal microbiota
IMT: Intestinal microbiota transplantation
qRT-PCR: Quantitative real-time PCR
PBS: Phosphate-buffered saline
PCoA analysis: Principal co-ordinates analysis
OTU: Operational taxonomic unit
LPS: Lipopolysaccharide
BA: Bile acid
7-ketoLCA: 7-Ketolithocholic acid
7-ketoDCA: 7-Ketodeoxycholic acid
UDCA: Ursodeoxycholic acid
LCA: Lithocholic acid
GCDCA: Glycochenodeoxycholic acid
CHO: Cholestyramine
GCRV: Grass carp reovirus
ISKNV: Infection spleen and kidney necrosis virus
IPNV: Infectious pancreatic necrosis virus
