# Correction: *Parabacteroides distasonis* regulates the infectivity and pathogenicity of SVCV at different water temperatures

**DOI:** 10.1186/s40168-024-01968-w

**Published:** 2024-11-18

**Authors:** Yujun Zhang, Yan Gao, Chen Li, Yong‑An Zhang, Yuanan Lu, Jing Ye, Xueqin Liu

**Affiliations:** 1 National Key Laboratory of Agricultural Microbiology, College of Fisheries, Huazhong Agricultural University, Wuhan, Hubei China; 2 Hubei Engineering Technology Research Center for Aquatic Animal Diseases Control and Prevention, Wuhan, Hubei China; 3 Ocean College, Hebei Agricultural University, Qinhuangdao, Hebei China; 4 Department of Public Health Sciences, Thompson School of Social Work & Public Health, University of Hawaii at Manoa, Honolulu, HI USA; 5 National Key Laboratory of Agricultural Microbiology, College of Veterinary Medicine, Huazhong Agricultural University, Wuhan, Hubei China

**Correction: Microbiome 12, 128 (2024)**

**https://doi.org/10.1186/s40168-024-01799-9**

Following publication of the original article [1], the author reported an error in Figs. 4E, 5G,5F and 6A.

Incorrect Fig. 4
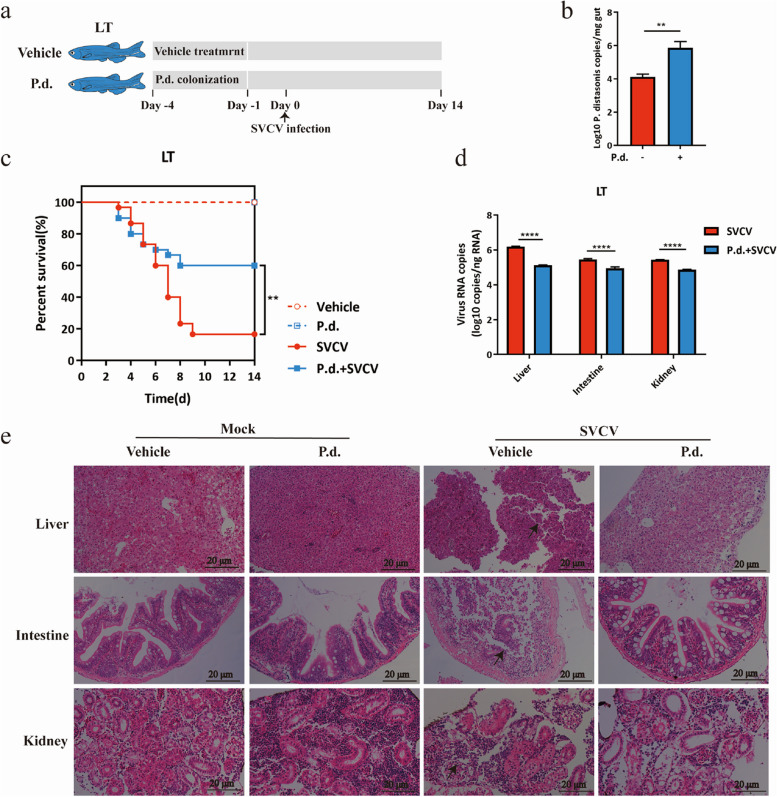


Correct Fig. 4
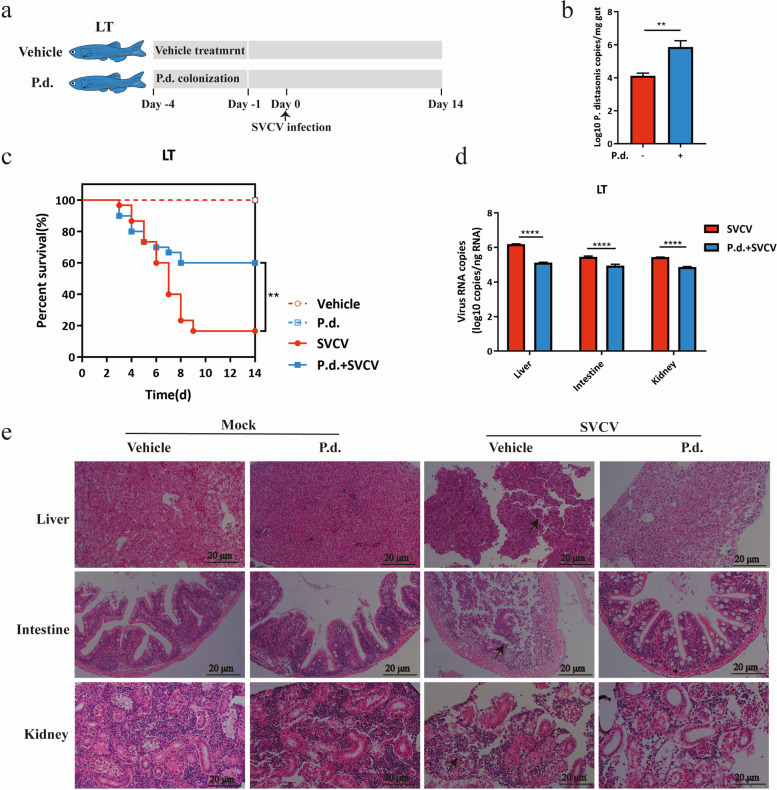


Incorrect Fig. 5
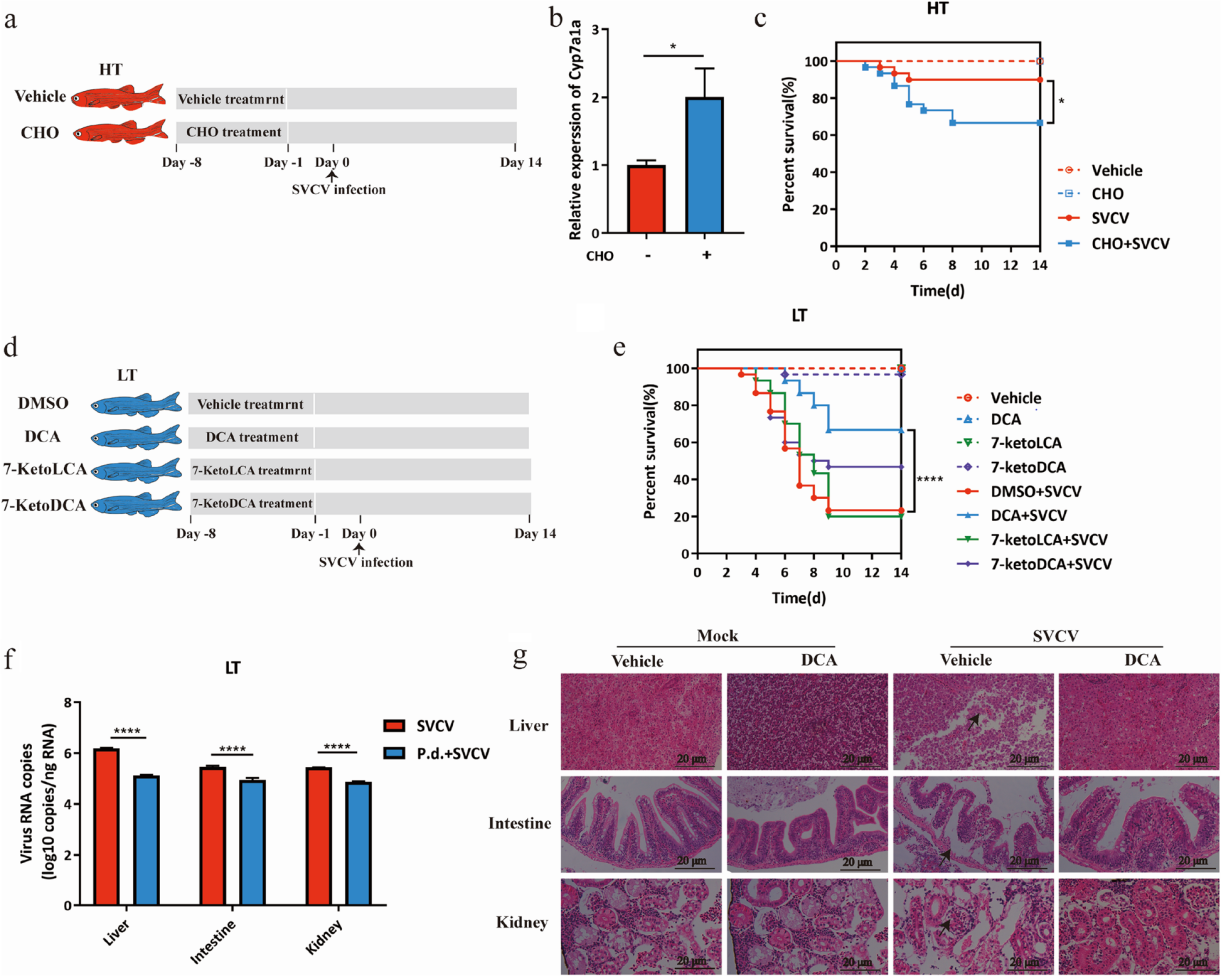


Correct Fig. 5
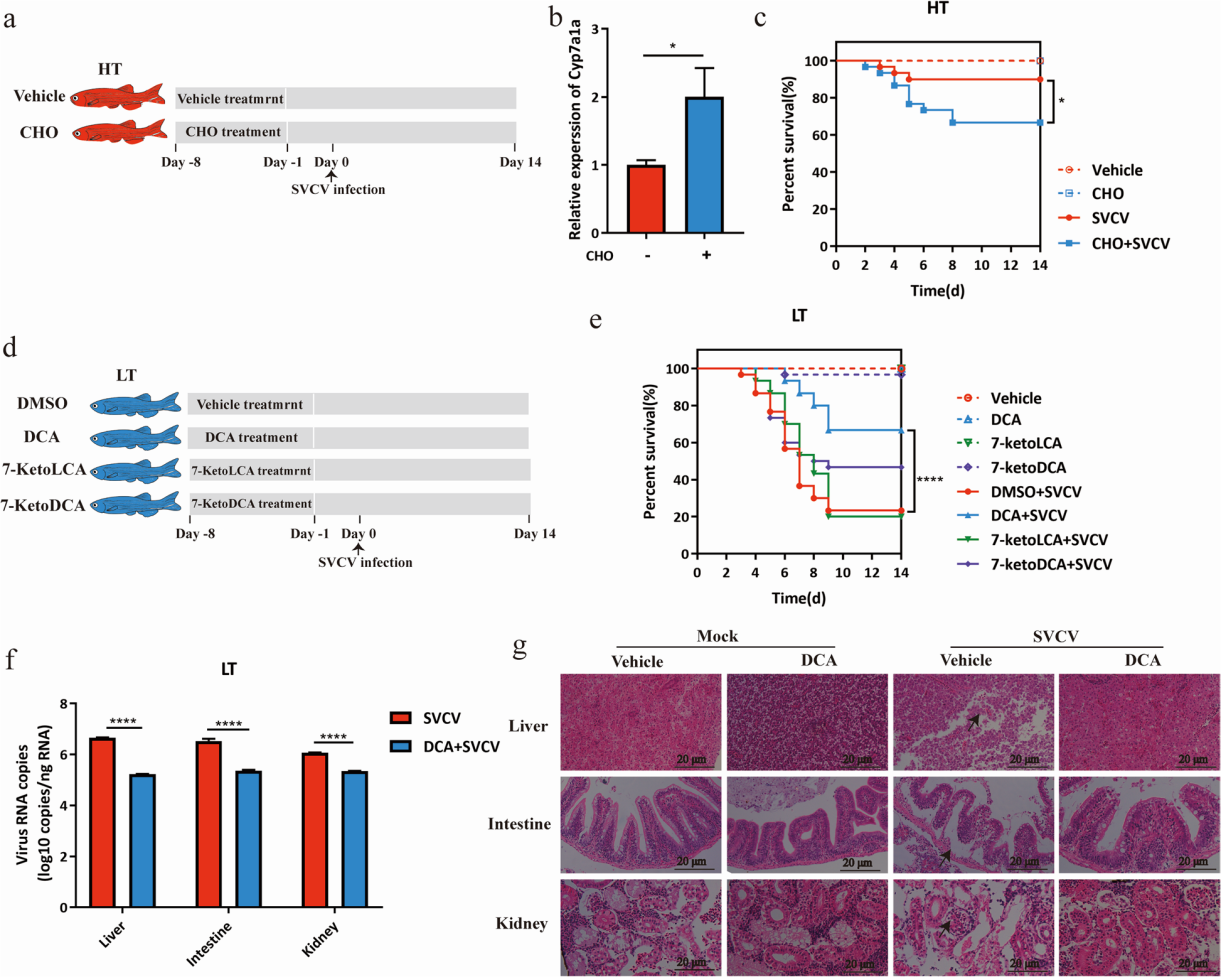


Incorrect Fig. 6
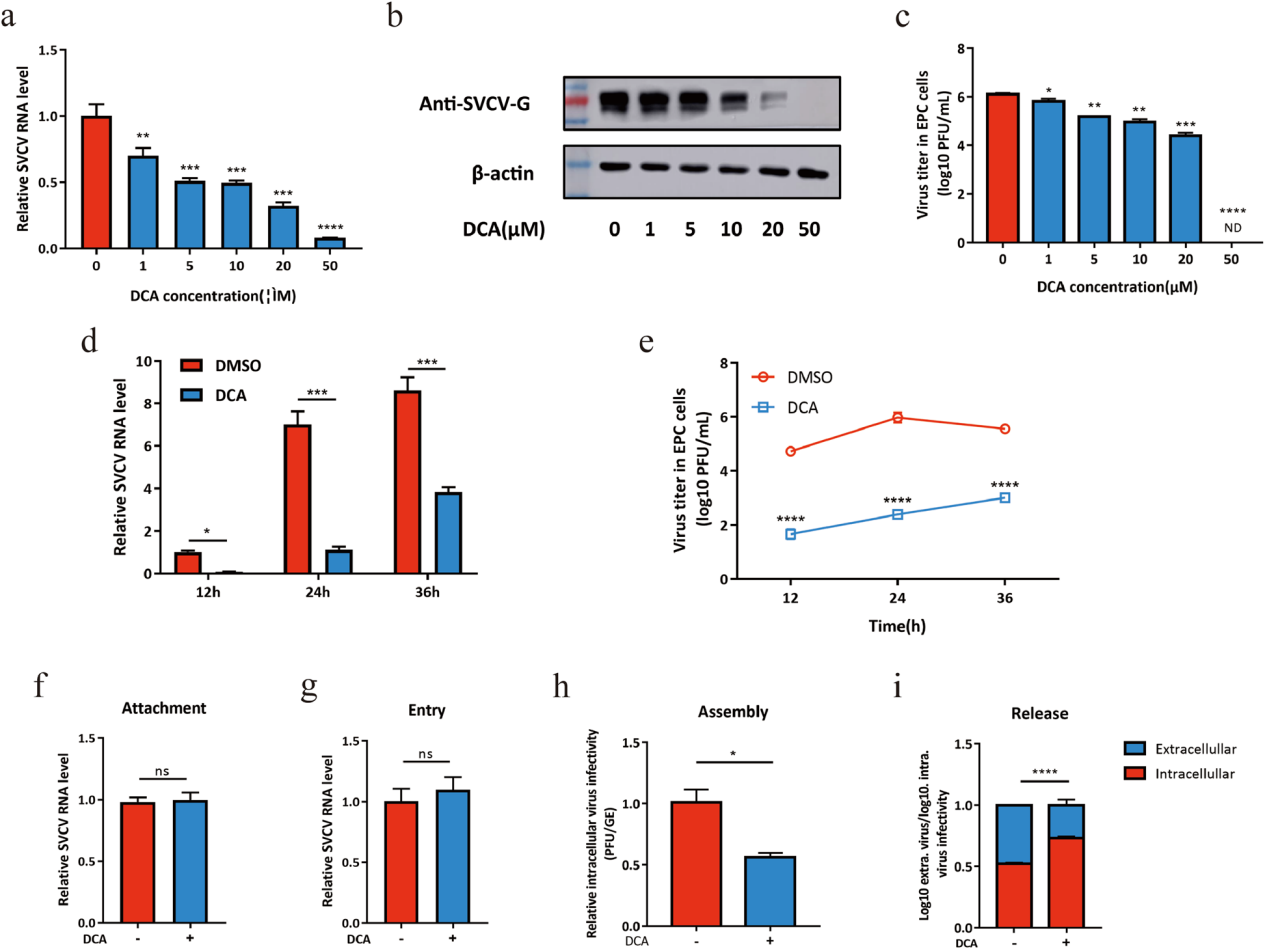


Correct Fig. 6
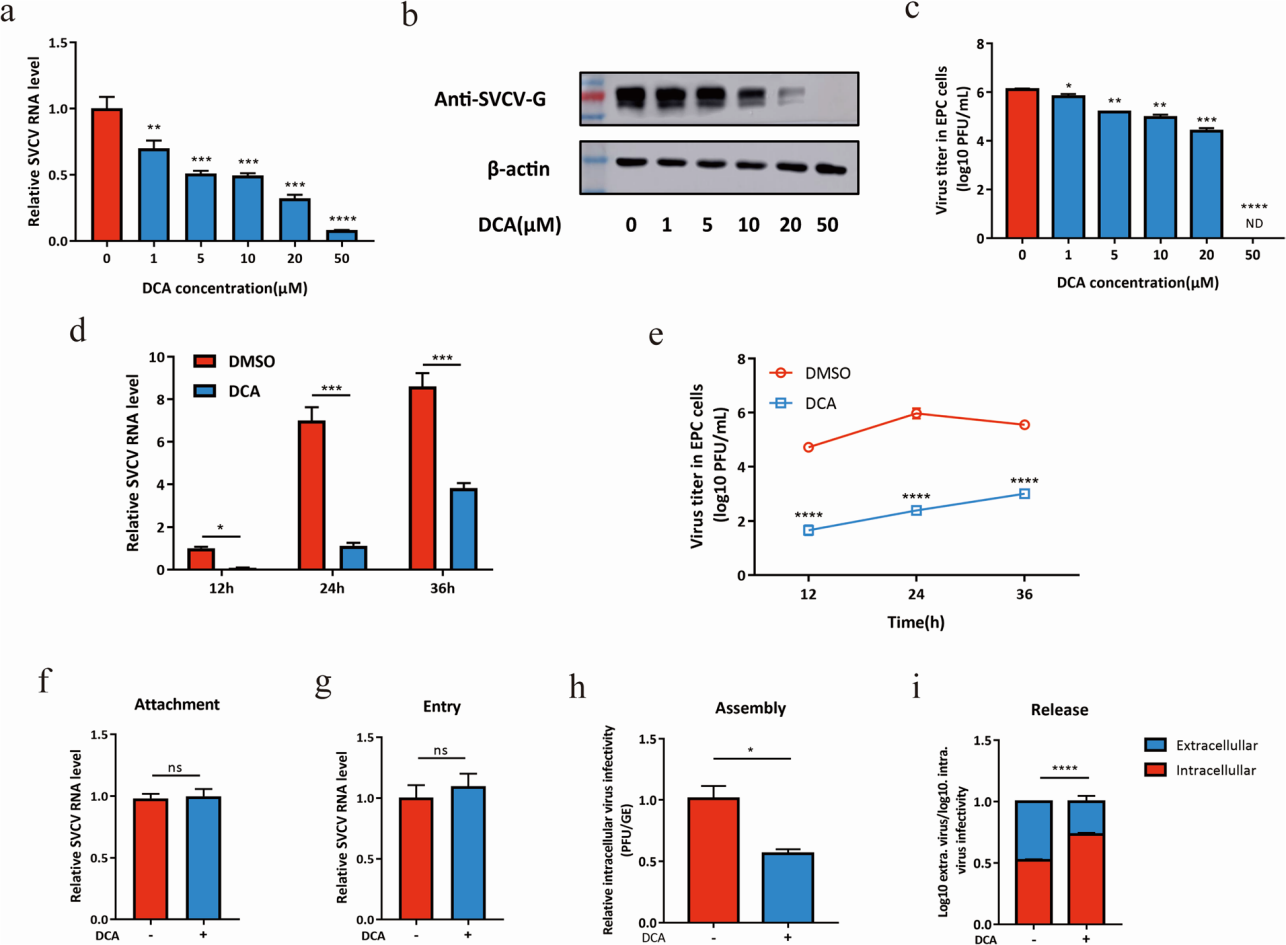


The original article has been updated.
