# Supplementary material for: Parabacteroides distasonis regulates the infectivity and pathogenicity of SVCV at different water temperatures
Source: Microbiome. 2024 Jul 17;12:128. doi: 10.1186/s40168-024-01799-9 (PMC11253412; doi:10.1186/s40168-024-01799-9)
Supplement: Supplementary file 2 — Additional file 1. Supplementary Figures S1-S2. [file 40168_2024_1799_MOESM1_ESM.docx]

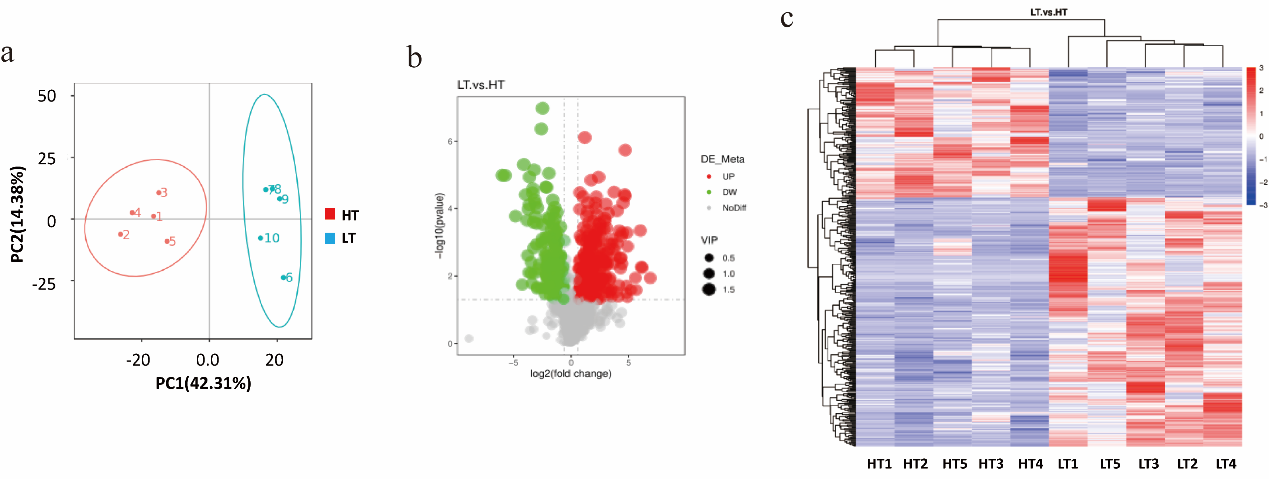


**Fig. S1. Characterization of the metabolomic profiles of HT and LT zebrafish**

**a** The metabolomic profiles were clustered using PLS-DA. The metabolomic profiles for the HT and LT groups are shown in the same colors. **b** The volcano map of differential metabolites. **c** Relative abundances of metabolites were clustered using a UPGMA dendrogram and shown in a heatmap. Color indicates the relative abundances of the metabolite in each sample; the corresponding relationship between the color gradient and the value is shown in the gradient color block.


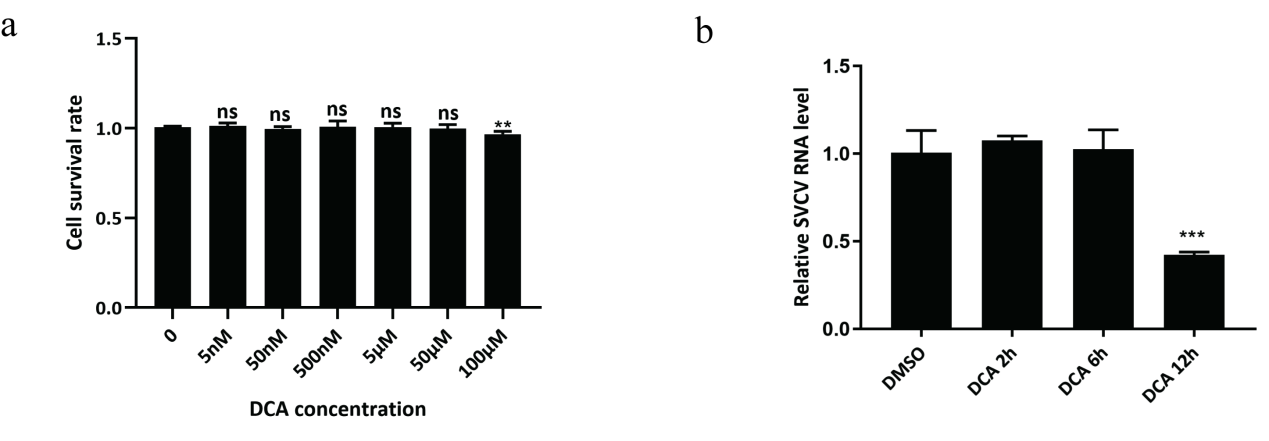


Fig. S2. **a** Cell viabilities of EPC cells were measured by a CCK-8 assay after these cells were incubated with DCA or DMSO for 48 h. **b** EPC cells were pretreated with DCA (10 μM) for 2h, 6h, 12 h and then infected with SVCV (MOI = 0.1). The RNA level of SVCV G was assessed by qRT-PCR analysis. All data shown are pooled from 2 independent experiments performed in triplicate (n=6, mean±SD).The statistics were analyzed using one-way ANOVA with Sidak’s post-test. *** *P* < 0.001.
