# Supplementary material for: Parabacteroides distasonis regulates the infectivity and pathogenicity of SVCV at different water temperatures
Source: Microbiome. 2024 Jul 17;12:128. doi: 10.1186/s40168-024-01799-9 (PMC11253412; doi:10.1186/s40168-024-01799-9)
Supplement: Supplementary file 3 — Additional file 2. Supplementary Tables S1. [file 40168_2024_1799_MOESM2_ESM.docx]

Table S1. Primers used for qRT-PCR expression analysis

| **Gene** |  | **Primer Sequence (5’-3’)** |
| --- | --- | --- |
| 40S | F | CCGTGGGTGACATCGTTACA |
|  | R | TCAGGACATTGAACCTCACTGTCT |
| P.d.-16s | F | GGTGGCATCCCTTTGTC |
|  | R | GTGCTGCATGGTTGTCGT |
| cyp7a1a | F | GCAGGCGTGCCAATGTG |
|  | R | CAGCTCGTTGAAGGTAGATAGTGTGT |
| Rt-actin | F | ATGGAAGGTGAAATCGCC |
|  | R | TGCCAGATCTTCTCCATG |
| IHNV-G | F | CACGGAAACAACACCACCATTA |
|  | R | AACAGCAAGGAGGAGAACAAGG |
| SVCV-G | F | TGCTGTGTTGCTTGCACTTATYT |
|  | R | TCAAACKAARGACCGCATTTCG |
|  | Probe | FAM-ATGAAGARGAGTAAACKGCCTGCAACAGA-TAMRA |
